# “Ecology of fear” in ungulates: Opportunities for improving conservation

**DOI:** 10.1002/ece3.8657

**Published:** 2022-03-01

**Authors:** M. Colter Chitwood, Carolina Baruzzi, Marcus A. Lashley

**Affiliations:** ^1^ 7618 Department of Natural Resource Ecology and Management Oklahoma State University Stillwater Oklahoma USA; ^2^ Department of Wildlife, Fisheries, and Aquaculture Mississippi State University Starkville Mississippi USA; ^3^ 3463 School of Forest, Fisheries, and Geomatics Sciences University of Florida Gainesville Florida USA; ^4^ 3463 Department of Wildlife Ecology and Conservation University of Florida Gainesville Florida USA

**Keywords:** antipredator behavior, predation risk, predator, prey, trait‐mediated effects, vigilance

## Abstract

Because ungulates are important contributors to ecosystem function, understanding the “ecology of fear” could be important to the conservation of ecosystems. Although studying ungulate ecology of fear is common, knowledge from ungulate systems is highly contested among ecologists. Here, we review the available literature on the ecology of fear in ungulates to generalize our current knowledge and how we can leverage it for conservation. Four general focus areas emerged from the 275 papers included in our literature search (and some papers were included in multiple categories): behavioral responses to predation risk (79%), physiological responses to predation risk (15%), trophic cascades resulting from ungulate responses to predation risk (20%), and manipulation of predation risk (1%). Of papers focused on behavior, 75% were about movement and habitat selection. Studies were biased toward North America (53%), tended to be focused on elk (*Cervus canadensis*; 29%), and were dominated by gray wolves (40%) or humans (39%) as predators of interest. Emerging literature suggests that we can utilize predation risk for conservation with top‐down (i.e., increasing predation risk) and bottom‐up (i.e., manipulating landscape characteristics to increase risk or risk perception) approaches. It is less clear whether fear‐related changes in physiology have population‐level fitness consequences or cascading effects, which could be fruitful avenues for future research. Conflicting evidence of trait‐mediated trophic cascades might be improved with better replication across systems and accounting for confounding effects of ungulate density. Improving our understanding of mechanisms modulating the nature of trophic cascades likely is most important to ensure desirable conservation outcomes. We recommend future work embrace the complexity of natural systems by attempting to link together the focal areas of study identified herein.

## Graphical abstract

In this review, we highlight the need to diversify research efforts on the ecology of fear in ungulates. We show that most of the literature found with our search disproportionately focused on a few prey and predator species, especially in North America, while several species and systems were highly underrepresented.

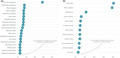

## 1 INTRODUCTION

The ecology of fear was conceptualized by Brown et al. (1999) as the “melding of the prey and predator's optimal behaviors with their population and community‐level consequences.” The ecology of fear concept synthesized the two approaches to predator–prey interactions (Hunter & Price, 1992; Paine, 1980; Peckarsky et al., 2008; Polis et al., 2000): (1) predators kill prey for food (Lima, 1998; Schmitz et al., 1997; Taylor, 1984); and (2) predators scare their prey (Lima & Dill, 1990; Peckarsky et al., 2008; Preisser et al., 2005; Schmitz et al., 2004; Trussell et al., 2006). These direct (i.e., lethal) and indirect (i.e., non‐lethal, non‐consumptive) effects of predation combine to affect prey and their interactions with the broader food web, which can generate indirect effects through processes in and across ecosystems (Hawlena & Schmitz, 2010a, 2010b; Hawlena et al., 2012; Peckarsky et al., 2008; Schmitz et al., 2010; Teckentrup et al., 2018).

The ecology of fear has gained momentum in recent years, having been applied to various terrestrial and aquatic systems (Dudeck et al., 2018; Michaud et al., 2016; Nunes et al., 2018). A plethora of literature has focused on ungulate responses to predation risk, likely because ungulates and their vertebrate predators are often charismatic (e.g., gray wolves [*Canis lupus*] and elk [*Cervus canadensis*]) and thus garner the most attention from a broad and diverse audience, particularly when set in well‐known locations (e.g., Yellowstone National Park, USA; African savannas). Moreover, ungulates are widespread globally, important economically and ecologically, and are often sympatric with large, apex predators that are of conservation concern (i.e., threatened, endangered, rare, reintroduced). Thus, broad review and understanding of the state of research into the ecology of fear is warranted, particularly given the interest in using the ecology of fear for conservation (Gaynor et al., 2021).

Recently, researchers have begun to summarize research topics related to the ecology of fear, including non‐consumptive effects of predation (Say‐Sallaz et al., 2019), the role of large carnivores in restoration ecology (Alston et al., 2019), methodological variation in characterizing predation risk (Moll et al., 2017), and improving inference in studies of predation risk (Prugh et al., 2019). Importantly, these studies are highlighting shortcomings and biases that could affect conservation and management decisions. For example, Say‐Sallaz et al. (2019) highlighted a strong taxonomic and geographic bias associated with research on non‐consumptive effects of predation in large terrestrial mammals, noting that gray wolves and North America dominated the peer‐reviewed literature. Likewise, they determined that antipredator behavioral responses of prey comprised the majority of the literature on non‐consumptive effects of predation (Say‐Sallaz et al., 2019). Other recent work highlighted a tendency among researchers to simplify otherwise complex systems by focusing on one carnivore and one ungulate when most systems being studied had multiple species of carnivores and/or ungulates (Montgomery et al., 2019). Study designs without experimental and longitudinal components likely oversimplify results and could be misleading or too general to be applied to other systems (Montgomery et al., 2019). Though these reviews identify biases that could affect large mammal conservation and management, none of them summarized the myriad research topics and results already published on ungulates under the ecology of fear concept. To address biases, improve future study designs on predation risk, and ultimately improve our understanding of how to use the ecology of fear in conservation, we sought to compile and summarize the current body of work from which future studies could develop increasingly complex questions into fear effects and their relevance to ecology, evolution, conservation, and management.

## 2 METHODS

**FIGURE 1:**
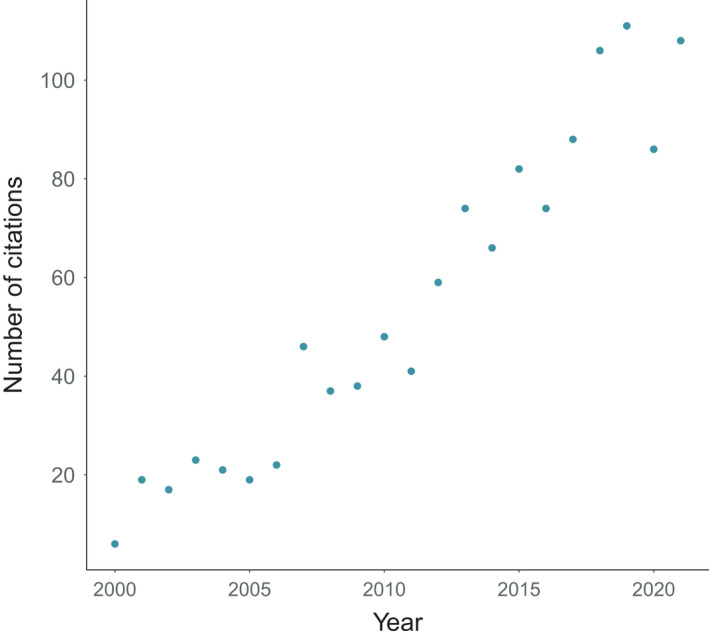
The number of citations per year (according to Google Scholar) of Brown et al. (1999), who conceptualized the ecology of fear

We conducted a literature search for articles using the keywords “ungulate” and “ecology of fear” or “landscape of fear” in the search engine Google Scholar. We purposely kept our search terms general to allow research themes to emerge from the published record, and we used each paper's literature cited to snowball sample other relevant work. We noted that most studies on ungulates and the ecology of fear fail to fully disentangle direct and indirect effects (Peers et al., 2018); however, we contend studies related to behavior and physiology are more well disentangled than those documenting other indirect effects in ecosystems. Thus, we considered the articles we found to be valuable research on the ecology of fear in ungulates, with the caveat that the mechanisms behind those effects may not be definitive in all articles cited. Also, though we refer to “ungulates” broadly throughout this paper, our focus was on ungulates for which fear‐based published work appeared in our search. Thus, we did not conduct searches for specific ungulate species (common or scientific names) or groups. We searched for studies between 1999 (when the ecology of fear concept was published) and July 2018. Additionally, we established a Google Scholar Alert that flagged papers indexed on Google Scholar after our search and before we completed our review of all the papers. This approach allowed us to include several papers published in 2018 and 2019, after the conclusion of our manual search. Though many ungulate‐focused predator–prey papers before 1999 could also be nested under the ecology of fear paradigm, we chose to focus on more recent literature where interest in the topic among academics has increased, as indexed by citations per year of Brown et al. (1999; Figure 1).

After surveying the literature, we grouped publications into four areas of focus, with some publications fitting under multiple categories. The areas of focus were behavioral responses to predation risk, physiological responses to predation risk, trophic cascades resulting from ungulate responses to predation risk, and manipulation of predation risk. We defined a behavioral response as any reactive or proactive response of ungulates to predation risks, including changes at fine‐scale (e.g., vigilance) or broad‐scale (e.g., habitat use). We defined physiological responses as any change in the physiology of ungulates as a result of predation risks, including changes in body chemistry (e.g., stress hormones) or disease risk. We defined a trophic cascade as occurring when predators altered ungulate behavior, resulting in the release of plants from herbivory (Polis et al., 2000); this could include any change in the plant community (i.e., plant distribution, abundance, or structure) resulting from the influence of predation risk on ungulates. Also, trophic cascades included cascading effects of ungulate responses to predation risk on other animal taxa or ecosystem processes. We defined manipulation of predation risk as any action taken or that could be taken by humans to intentionally cause fear (i.e., increasing perceived or actual predation risk via top‐down or bottom‐up management approaches) to evoke a desirable ecological consequence.

## 3 RESULTS AND DISCUSSION

**FIGURE 2:**
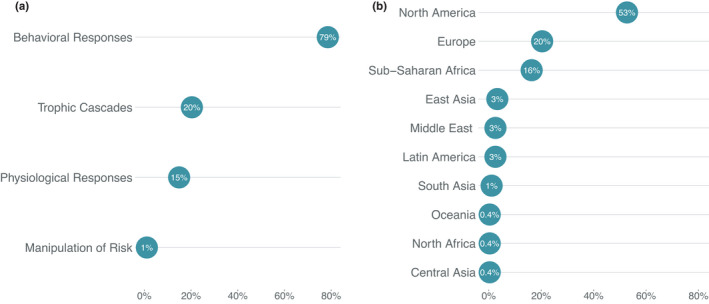
Proportion of research papers focused on each of four major topic areas of study (a) and proportion of research papers by geographic area of focus (b). Because papers could cover multiple topics, proportions in A do not sum to 1

**FIGURE 3:**
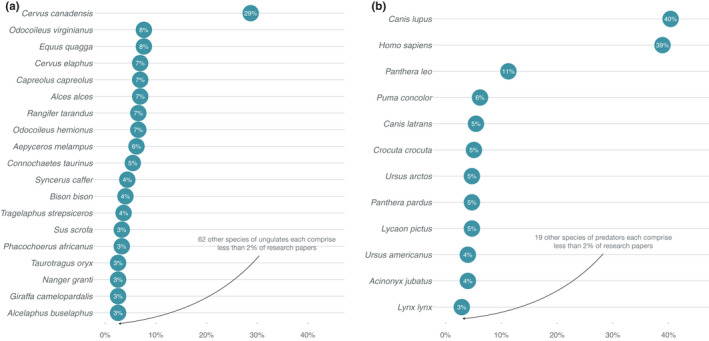
Proportion of research papers focused on different ungulate taxa (a) and proportion of research papers focused on different predator taxa (b). Because papers could include multiple ungulate and predator taxa, proportions do not sum to 1

The literature search yielded 275 studies relevant to the ecology of fear in ungulates (Appendix S1). While most papers covered multiple topics, the most studied area of focus was behavioral responses to predation risk (e.g., habitat selection, space use, vigilance; 79%; *n* = 216; Figure 2a). Some studies were focused on trophic cascades (20%; *n* = 56), while fewer focused on physiological effects of fear (15%; *n* = 41) and only three (1%) on manipulation of predation risk for wildlife management (Figure 2a). More than half of the studies took place in North America (53%; *n* = 145; Figure 2b), mainly in the Greater Yellowstone Ecosystem (*n* = 60; 22% of all studies; 41% of North American studies). Fewer studies were conducted in Europe (20%; *n* = 56), Sub‐Saharan Africa (16%; *n* = 45), and other world regions (11%; *n* = 29; Figure 2b). Overall, 81 ungulate species were studied since the fear concept was first published, with studies of elk comprising the largest proportion (29%; *n* = 79; Figure 3a). The majority of research was focused on just a few predators, dominated by gray wolves (40%; n = 111; Figure 3b) and humans (39%; *n* = 107; Figure 3b) that together accounted for 79% of the studies (*n* = 218).

### 3.1 Behavioral responses to predation risk

In the presence of predators, prey generally alter their behavior to become more difficult to capture, detect, or encounter. Antipredator behaviors are a complex suite of innate and learned behavioral responses, which can be individual or species‐specific (Chamaillé‐Jammes et al., 2014; Thurfjell et al., 2017). They can be affected by predator species and habitat characteristics. For example, ambush predators make animals more fearful of complex vegetative structure with poor visibility likely because of uncertainty in the predator location (Lone et al., 2014), whereas cursorial predators make animals more fearful of areas with high visibility and poor escapability (Riginos & Grace, 2008; Ripple & Beschta, 2003, 2006a). Additionally, human activities can elicit fearful responses in ungulates and, in human‐dominated landscapes, human presence and activity can affect ungulate behavior and predator–prey dynamics (Ciuti et al., 2012; Shannon et al., 2014). Human hunting could oppose adaptive responses to natural and sexual selection through exploitation‐induced evolutionary change (Ciuti et al., 2012). We separated the studies of ungulate behavior in response to predation risk (*n* = 216) into two subtopics: movement and habitat selection (75%; *n* = 161) and vigilance and herding (32%; *n* = 70).

#### 3.1.1 Movement and habitat selection

Habitat quality is important to how ungulates reduce predation risk (Bleicher, 2017). In fact, animals can mitigate predation risk in various ways such as reducing the time spent foraging, foraging in less risky areas or at less risky times, or increasing vigilance when foraging in risky places (Brown, 1999; Gehr, Hofer, Ryser, et al., 2018). In this way, animals move around the landscape adjusting their behavior to accommodate spatiotemporal variation in predation risks (Basille et al., 2015).

Spatial avoidance is commonly reported in ungulates to reduce predation risk, but less work has documented temporal changes to avoid risk. Several species such as mule deer (*Odocoileus hemionus*; Laundré, 2010), elk (Bacon & Boyce, 2016; Fortin et al., 2005), and hartebeest (*Alcelaphus buselaphus*; Ng’weno et al., 2017) exhibit a negative relationship in spatial distribution with predation risk. However, avoidance can be mediated by resource availability. For example, hartebeest, plains zebra (*Equus quagga*), and Grant's gazelle (*Nanger granti*) prefer areas with high grass biomass to areas of high visibility during droughts (Riginos, 2015). A study of activity patterns in Sunda clouded leopard (*Neofelis diardi*) shows that in the absence of clouded leopards, bearded pigs (*Sus barbatus*) were more nocturnal than when leopards were present, perhaps indicating the bearded pigs alter their activity pattern to decrease predation risk (Ross et al., 2013). One study looked at roe deer (*Capreolus capreolus*) spatial and temporal behavior reporting that roe deer avoid areas of high chronic predation by Eurasian lynx (*Lynx lynx*) at night but not during the day in summer because lynx activity is low due to human disturbance during the day (Gehr, Hofer, Pewsner, et al., 2018).

The decision of where and when to forage or seek cover occurs across spatiotemporal scales (Lima & Dill, 1990) and even small habitat changes can play an important role in prey habitat selection because they affect prey cost of locomotion (Gallagher et al., 2017). Altendorf et al. (2001) concluded that mule deer respond to predation risk from mountain lions (*Puma concolor*) by changing their foraging decisions at the scales of vegetation types and specific features of the vegetation type such as edges. At finer scales, many studies have documented behavioral responses to predation risk related to forage selection and quality. For example, bison (*Bison bison*) reduced selection of high‐quality foraging sites (i.e., sites with abundant *Carex atherodes*) as wolf risk increased in winter (Fortin & Fortin, 2009). Hamel and Côté (2007) reported that female mountain goats (*Oreamnos americanus*) traded off forage abundance (and some forage quality) for safety cover. Similarly, some studies have linked behavioral effects of predation risk to fine‐scale landscape features and vegetative cover. For example, Nubian ibex (*Capra nubiana*) perceived greater risk of predation as their distance from cliff and slope edges increased, and their perception of risk decreased with vegetative cover (Iribarren & Kotler, 2012). Likewise, Kuijper et al. (2013) linked coarse woody debris to fine‐scale risk effects on ungulates in the presence of wolves.

Movement, space use, and habitat selection also likely relate to predator hunting mode. For example, a study in South Africa (Thaker et al., 2011) using seven ungulates and five large carnivores determined that most of the smaller prey species (e.g., impala [*Aepyceros melampus*]) avoided the space use of all predators to reduce probability of encounter. Concomitantly, larger species (e.g., blue wildebeest [*Connochaetes taurinus*]) only avoided areas of intense space use by lions (*Panthera leo*) and leopards (*Panthera pardus*). The authors concluded that ungulates used a simple behavior rule: avoid areas used by sit‐and‐pursue predators (lion and leopard) but increase wariness in areas used by cursorial predators (e.g., cheetah [*Acinonyx jubatus*] and African wild dog [*Lycaon pictus*]). Similarly, other studies using predator excrement at foraging areas monitored with camera traps demonstrated red deer (*Cervus elaphus*) were not only apparently able to discern hunting mode from the type of excrement present, but also used different antipredator behaviors to mitigate risk of each threat (Wikenros et al., 2015). Red deer spent less time foraging at sites when threatened by ambush‐style predation risk but only adjusted vigilance under cursorial‐style predation risk. Multiple decision rules combine to affect ungulate space use (and other antipredator behaviors), especially in multi‐predator systems where predators differ in hunting mode (Thaker et al., 2011).

Some studies have reported weak evidence for behavioral responses to predation risk. For example, Nicholson et al. (2014) found little support that moose (*Alces alces*) habitat use was dependent on predation risk from wolves, though they acknowledged several underlying explanations that could have been confounding (i.e., intense harvest by humans, no time to adapt to recolonizing wolves, adaptation may occur at finer scales than measured). Similarly, Samelius et al. (2013) concluded that recolonizing lynx (*Lynx lynx*) had limited effects on habitat selection of roe deer (*Capreolus capreolus*) in Sweden. The authors suggested their results provided evidence for the complexity of prey responses to risk and that such responses likely were variable between ecosystems and predator–prey constellations (Samelius et al., 2013). Results from Hernández and Laundré (2005) may support this premise, as they concluded that predation pressure from reintroduced wolves shifted elk habitat use thereby decreasing their diet quality but did not result in a similar change in space use or diet quality of bison. The weak evidence for behavioral responses to predation risk in these studies, coupled with differing responses of sympatric ungulates, may be linked to predator hunting mode (Thaker et al., 2011), antipredator strategies of the ungulates (Eby et al., 2014), size discrepancies between predator and prey (Eby et al., 2014), a lack of a response, or failure to detect it with study design or sample size.

One interesting behavioral concept that relates to movement and habitat selection is the idea that ungulates are using intraguild interactions to mediate the landscape of fear by concentrating activity in proximity to humans as a shield to other predators (Berger, 2007; Schmitz et al., 2004). Because humans are predators of ungulates, situations where humans are used as shields to other predators represent an interesting twist, whereby ungulates apparently perceive humans as less threatening than other predators. Thus, ungulates may actually use a carnivore's fear of humans to their own benefit. For example, Berger (2007) documented synchrony in moose parturition, which involved changes in moose space use commensurate with carnivore recolonization. Mothers in areas free of brown bears (*Ursus arctos*) and non‐parous females did not alter space use, while those giving birth did so nearer to paved roads avoided by brown bears (Berger, 2007). Similarly, mule deer females appear to compensate for greater exposure to predation risk by increasing their activity and herbivory intensity close to a remote biological field station, presumably because they could forage more selectively in areas coyotes avoided due to human activity (Waser et al., 2014). Such results indicate that shifts in space use likely have occurred in other mammalian taxa in the presence of humans and that researchers should account for indirect anthropogenic effects on species distributions, behavior, and interactions (Berger, 2007). Fear of the human “super predator” may be relevant for large carnivores (Smith et al., 2017) and ungulates (Crawford et al., 2022) alike, but the extent to which such fear varies across landscapes and taxa is unknown. For example, predators such as coyotes may be resilient to urbanization, and thus, initiate even more complex predator–prey interactions in urban areas (Jones et al., 2016).

#### 3.1.2 Vigilance and herding

Vigilance of prey species is one of the most studied aspects of antipredator behavior because it is one of the most common adaptations used by animals for evaluating predation risk and is relatively easy to measure (Benoist et al., 2013). Time spent scanning for predators generally prevents animals from other activities (but see Périquet et al., 2012 and Bergvall et al., 2016), such as foraging or grooming, so that animals must carefully trade between reducing risk and acquiring energy (Creel, 2018; Illius & Fitzgibbon, 1994). The amount of time allocated in vigilance depends on risk perception. For instance, Dröge et al. (2017) show that African ungulates (i.e., hartebeest, plain zebra, and oribi [*Ourebia oribi*]) increase vigilance when close to predators in places where predator encounter probability is high. Vigilance also depends on herd size because herding ungulates generally rely on group vigilance so that individuals can spend less time scanning for predators as group size increases (Lima & Dill, 1990). As such, herd size is also related to risk perception. For instance, Moll et al. (2016) reported that herd size in several African ungulate species depends on predator hunting mode and duration of predation risk. However, vigilance and herd size are not always directly related, as they also depend on other factors affecting individual risk such as reproductive status (Li et al., 2012), sex (Barnier et al., 2016; Benoist et al., 2013), offspring presence (Blanchard et al., 2017; Lashley et al., 2014), intraspecific competition (Biggerstaff et al., 2017; Fattorini et al., 2018), habitat features (Pays et al., 2012), cover and visibility (Iranzo et al., 2018; Pays et al., 2012), prey foraging strategy (Creel et al., 2014), and predator presence (Iranzo et al., 2018).

We still do not fully understand the nuances of antipredator behaviors like vigilance and herding (Beauchamp, 2019). For example, Creel et al. (2008) determined that Gallatin elk were more vigilant than Northern Range elk despite lower background risk in the Gallatin Canyon. Indeed, Le Saout et al. (2015) provided evidence that vigilance behavior probably persists at some level, even in the absence of predation risk. Presumably, the costs associated with overt vigilance are too low in some cases to generate strong selection pressure for non‐vigilant phenotypes, particularly given the consequences of being unequipped to avoid predation in the future (Le Saout et al., 2015). Likewise, the level of risk may interact with group size to affect vigilance response in some cases but not in others, and vigilance may also be used to monitor conspecifics, especially in low‐risk situations (Beauchamp, 2019). Olfactory and auditory cues are used to assess relative risk, but they are also understudied (Lynch et al., 2014). For example, the odor of wolves and lynx can create fine‐scale risk factors for red deer (Kuijper et al., 2014; Wikenros et al., 2015). As noted earlier, red deer apparently discern between the predator hunting mode based on odors from excrement, adjusting their antipredator strategy accordingly (Wikenros et al., 2015). However, our understanding of how olfactory and auditory cues are used in avoiding predation risk is rudimentary, and we need further research to evaluate the use of olfactory cues in different species.

### 3.2 Physiological responses to predation risk

Ungulates must balance forage acquisition and risk avoidance, which necessitates interplay between physiology and behavior (McArthur et al., 2014). A scant amount of research goes even further, likening ungulate physiological responses to parasitism and disease to the responses documented under fear of predation. Behavior, as discussed in the previous section, is an interface that enables ungulates to use or leave forage patches depending on their physiological tolerance to risk (McArthur et al., 2014). By default, these behavioral choices in response to predation risk can be related to diet quality and nutritional costs, and we chose to include diet quality and nutrition in this section, while recognizing that they are topics arguably sorted into “behavior” as well. We separated studies on ungulate physiological responses to predation risk (*n* = 41) into two subtopics: diet quality and nutrition (71%; *n* = 29) and fitness and physiology (32%; *n* = 13).

#### 3.2.1 Diet quality and nutrition

Behavioral responses adopted by prey species under threat of predation induce important risk effects on the prey, especially nutritionally‐mediated risk effects. As previously mentioned, prey may switch to lower quality food patches if risk is decreased enough to warrant the cost to foraging or ungulates may reduce their food intake to increase vigilance. For example, plains zebras in close proximity to lions had a lower quality diet, indicating that adjustments in behavior when near lions carry nutritional costs (Barnier et al., 2014). White‐tailed deer (*O*. *virginianus*) switched to an abundant low‐quality food (i.e., oak *Quercus* spp.) in response to stress from coyotes (Cherry, Warren, et al., 2016). Similarly, predation pressure from reintroduced wolves in the Greater Yellowstone Ecosystem induced shifts in elk habitat use, which lowered the quality of the elk diet (Hernández & Laundré, 2005). However, nutritionally‐mediated risk effects are not necessarily ubiquitous in all predator–prey relationships, as Hernández and Laundré (2005) also reported that bison did not display a similar change in habitat use and dietary quality to what they observed in elk.

An emerging literature base also indicates that predation risk can cause physiological changes that alter the perceived relative importance of nutrients, which may affect dietary choices and health (Hawlena & Schmitz, 2010b). This has been well demonstrated in an arthropod system where spiders change diet selection of prey by changing its physiological demands for carbohydrates (Barton, 2010; Beckerman et al., 1997; McMahon et al., 2018; Rothley et al., 1997; Schmitz, 1998). Interestingly, similar results have been reported in vertebrate taxa (Carmassi et al., 2015; Clinchy et al., 2013; Klingaman et al., 2016; Leaver & Daly, 2003), but examples from ungulates have not been reported. Although demonstrating predation risk inducing physiological changes that manifest in health and behavior is inherently difficult, this new frontier of merging nutritional ecology with predation risk theory has the potential to advance our understanding of the ecology of fear.

#### 3.2.2 Fitness and physiology

Boonstra (2013) suggested that several ungulate species that evolved with large predators are adapted to coping with predation pressure and therefore they suffer from acute stress (i.e., elevated glucocorticoids blood level for minutes to hours). On the contrary, other mammal species such as snowshoe hare or arctic ground squirrel may suffer from chronic stress showing elevated chronic (i.e., days to weeks) glucocorticoids blood level, which may have negative fitness consequences (Boonstra, 2013), even for future generations (Sheriff et al., 2010). Some research investigating glucocorticoid stress hormones studying ungulates reported similar patterns (Creel et al., 2009; Le Saout et al., 2016; Pecorella et al., 2016; Périquet et al., 2017; but see Zwijacz‐Kozica et al., 2013), but further investigation on ungulate hormonal response to predation and its fitness consequences are needed. In fact, predation has been related to decreased fecundity in hartebeest (Ng’weno et al., 2017) and white‐tailed deer (Cherry, Morgan, et al., 2016, but see Michel et al., 2020) and contrasting results have been reported in elk (Creel et al., 2011; Middleton et al., 2013). Predator‐induced stress and selection of low‐quality forage to avoid predation have been suggested to cause decreased fecundity (Christianson & Creel, 2010; Ng’weno et al., 2017), but the specific pathways through which predation indirectly affect individual fitness still have to be defined.

### 3.3 Trophic cascades resulting from ungulate responses to predation risk

Ungulates represent the intermediate trophic level, potentially linking apex predators to changes in plant communities. Thus, trophic cascades are caused via behavioral adjustments and density responses of ungulates to predation risk that, in turn, affect the distributions and relative abundances of plants and may indirectly affect other biota and ecological processes as well (Beschta & Ripple, 2009; Estes et al., 2011; Ripple & Beschta, 2012; Ritchie & Johnson, 2009). Two types of trophic cascades have been described in the literature: (1) density‐mediated, and (2) trait‐mediated (Werner & Peacor, 2003). Density‐mediated trophic cascades occur as a result of ungulate population regulation by apex predators, which release palatable plant species from herbivory. Trait‐mediated trophic cascades result from ungulate antipredator behaviors in response to the perception of predation risks that are invoked by predators (see previous sections on behavior and physiology for examples of specific trait modifications).

Trait‐mediated trophic cascades could release plants from herbivory due to spatial avoidance or decreases in foraging rate due to predator presence (Ripple et al., 2016). Studies on trait‐mediated trophic cascades generally entail systems with a single prey and predator, which creates a knowledge gap regarding trophic cascades in more diverse predator or prey contexts (Flagel et al., 2016; Ripple et al., 2015). Many historical ecosystems had multiple predators with each hunting mode, making the behavioral decisions of the ungulate more complicated and the resulting trophic cascade presumably more complex; thus, the tri‐trophic cascade generally studied might not represent all complex situations (Norum et al., 2015; Schmitz et al., 2004; Thaker et al., 2011). For example, Ford et al. (2015) reported that the reintroduction of African wild dogs (*Lycaon pictus*) suppressed dik dik (*Madoqua guentheri*) populations but did not result in trophic cascades to the plant community, likely because of herbivore diversity in the system. Furthermore, surrogate predators, either introduced or invading, may or may not cause trait‐mediated trophic cascades similar to that of native predators, even if they have the same general hunting mode. As an example, coyotes have recently expanded their range across eastern North America (Hody & Kays, 2018), and studies in the southeastern United States have implicated them as an important predator and primary cause of sharp population declines of white‐tailed deer in some areas (Chitwood et al., 2014; Chitwood, Lashley, Kilgo, Moorman, et al., 2015; Chitwood, Lashley, Kilgo, Pollock, et al., 2015; Kilgo et al., 2012). Though they are coursing predators similar to the primary historical predator (i.e., red wolf [*Canis rufus*]), recent literature has reported coyote selection against behavioral traits of white‐tailed deer that had presumably evolved as an adaptive response to red wolves (Chitwood et al., 2017). Moreover, coyotes are more resilient than wolves to urbanization, so they may exert greater controls on ungulates in urbanized landscapes (Jones et al., 2016). That said, coyotes can have cascading effects on plant communities by altering traits of white‐tailed deer (Cherry, Warren, et al., 2016). Considering the rapidly changing climate and burgeoning human urbanization, the expectations of predators expanding ranges into new areas is realistic and the effects of new predators and new predator–prey contexts may become an increasingly important area of focus. Indeed, trait‐mediated trophic cascades can be mediated by several potentially interacting factors, leading to debate on the actual existence of the trophic cascades. Many observations have been scrutinized and contrasting results have been presented (Creel & Christianson, 2009; Kauffman et al., 2010), bringing into question whether or not trait‐mediated indirect effects are important parts of ecosystems or rather just the result of research failing to disentangle them from density‐mediated mechanisms.

Predicting the strength of trophic cascades (i.e., how far they reach across taxa and ecological processes, as well as the magnitude of their effects) is complicated because a multitude of factors affects this phenomenon (Schmitz et al., 2004). Shurin and Seabloom (2005) reported the strength of cascades was related to size discrepancy between herbivores and plants, whereas predator body size in relation to the ungulate had no effect. Contrastingly, DeLong et al. (2015) reported that predator body size was important in determining the strength of resultant trophic cascades because the strength of predator‐prey interactions generally increases with predator size. Also, predator density might be important in the strength of the resulting trophic cascades. For example, Beschta and Ripple (2010) reported the reintroduction of Mexican wolves (*C*. *lupus baileyi*) did not result in a trophic cascade on aspen in Arizona, perhaps because the density of wolves was too low relative to elk densities (i.e., 3 wolves per 100 elk).

There are three ways trophic cascades are generally studied: (1) predator removal or exclusion, (2) predator reintroduction, and (3) ungulate exclusion (Shelton et al., 2014). The first two methods are fundamentally different in that predator removals are measuring the trophic cascades leading to what is considered ecological degradation (Côté et al., 2004), and predator reintroductions are measuring trophic cascades presumed to be leading to ecological restoration (Ripple & Beschta, 2004). The third approach (i.e., ungulate exclusion) may study trophic cascades from either point of view, and the methods may be paired to yield stronger inferences (Ford & Goheen, 2015).

#### 3.3.1 Predator removal

Predator removal experiments have been conducted to measure the cascading effects in many systems dominated by avian, lizard, and ant predators (Schmitz et al., 2000). However, large predator removal experiments are more difficult to control at the scale needed to study ungulate systems. The widespread extirpation of apex predators has given rise to several opportunities, albeit usually with poor replication, to study how ungulates affect ecosystems without predation risks (Ritchie et al., 2012). In systems without predators, ungulate populations may increase substantially, degrading the plant community as a result of intense unimpeded herbivory (Côté et al., 2004). Several examples exist to corroborate this notion. Berger et al. (2001) found that the loss of grizzly bears and gray wolves led to the degradation of riparian areas via density‐mediated moose herbivory, which eroded the bird community in the Greater Yellowstone Ecosystem. Ripple and Beschta (2006a) reported a density‐mediated trophic cascade linking increased human presence to cougar declines, increased mule deer density, decreased cottonwood regeneration, increased soil erosion, and decreased aquatic and terrestrial diversity in Yellowstone National Park. Likewise, Wallach et al. (2010) reported that predator control of dingoes (*C*. *lupus dingo*) resulted in population increases in invasive herbivores and decreases in biodiversity. Finally, in a review, Estes et al. (2011) detailed many trophic cascades through different trophic levels and ecological processes resulting from the extinction of apex predators, including alterations of disease dynamics, wildfire on the landscape, carbon sequestration patterns, invasive species invasions and prevalence, and biogeochemical cycles.

Interestingly, recent evidence has indicated that ungulate densities may exceed nutritional carrying capacity for decades without nutritional feedback on the population (Le Saout et al., 2014). That same research also highlights the disparity between stable states of ungulate populations with and without predators and how drastic alternative stable states in ungulate populations may affect ecosystem processes. The extensive herbivory pressure may result in natural selection favoring plant species with heightened herbivory defenses (Strauss & Agrawal, 1999) or induce plant defenses within species (Stotz et al., 2000). However, top‐down controls likely will limit vertebrate populations to a lower density than bottom‐up controls, creating the disparity in stable states often observed between predator and predator‐free environments (Terborgh et al., 2001).

#### 3.3.2 Predator addition

The reintroduction of wolves to Yellowstone National Park has provided the standard example of how fear affects ungulates in ways that cascade to plant communities, dependent wildlife species, and other ecological processes (Beschta & Ripple, 2009; Estes et al., 2011; Ripple & Beschta, 2006b, 2012; Ritchie & Johnson, 2009). We commonly think of the scenario as restoring ecosystem function because the predator reverts ungulate populations and behavior from the alternative stable state back to the historical stable state. These “natural experiments” provide the opportunity to evaluate the resilience of an ecosystem to the alternative stable state because we can observe the recovery of ecological processes. For example, Ripple and Beschta (2003) monitored cottonwood recovery following reintroduction of wolves and noted that riskier sites had taller trees and greater annual growth, and height was significantly correlated to gully depth, which is linked to escapability or riskiness of the area. Those areas were most susceptible to herbivory consequences following the extirpation of wolves but also were more resilient because of a faster recovery time. The reintroduction of predators may provision other ecosystem services that are not readily anticipated. For example, wolves affect grazing by ungulates in ways that cascades to altered microbial activity and nutrient dynamics of grasslands (Frank, 2008). Ripple et al. (2014) reported another example where wolf presence modulated grizzly bear diet indirectly by affecting fruit production through the regulation of elk density and foraging behavior. Even the geomorphology of rivers may be affected by herbivory differently depending on whether the ungulates are foraging under the risk of predation (Beschta & Ripple, 2012).

#### 3.3.3 Ungulate exclusion

Ungulate herbivory can have ecosystem wide and long‐term consequences. For example, Nuttle et al. (2011) demonstrated in a long‐term ungulate exclusion experiment that high white‐tailed deer density at stand initiation resulted in century‐long changes in ecosystem function, including simplified forest structure and composition, decreased canopy foliage density, decreased insect diversity, and decreased bird diversity. Similarly, Shelton et al. (2014) used ungulate exclosures to show that white‐tailed deer had cascading effects on plant communities in all forage classes, which indirectly affected small wildlife species. Ford et al. (2015) reported that the recovery of wild dogs following reintroduction in Kenya limited densities of dik dik but did not trigger a trophic cascade, possibly because of the diversity of browsers or a time lag in indirect effects.

### 3.4 Manipulation of predation risk

Recently, researchers and practitioners have come to the realization that management strategies can potentially use fear of predation as a basis for management decisions (Cromsigt et al., 2013; Suraci et al., 2016). Indeed, humans have used fear to deter wildlife damage since the dawn of agriculture. For example, the use of a scarecrow is commonplace and serves as a visual cue to ward off depredating wildlife in crop fields. Likewise, farmers have recommended the use of human hair as a scent cue to deter deer from gardens. These household remedies for depredation by ungulates are rooted in the ecology of fear concept and provide classic examples of how the landscape of fear can be manipulated as a management tool. Generally, the landscape of fear can be managed by passive (e.g., predator reintroductions) and active (e.g., hunting, predator cues, habitat manipulation) means, with a top‐down or bottom‐up approach.

#### 3.4.1 Top‐down approaches

Berger et al. (2001) suggested the potential for using human hunting to invoke the trophic cascades provided by wolves to restore ecosystem function. Cromsigt et al. (2013) embraced this idea with the concept of “hunting for fear,” where they proposed using hunting as a top‐down approach to managing undesired effects of ungulates. Other potential methods such as training domesticated predators to deter prey (Atkins et al., 2017) are being used increasingly to mitigate human‐wildlife conflicts, but these likely are less practical for ungulates. An important consideration when designing and studying these management approaches is how the natural apex predator of the system affects ungulate behavior and how the resulting behaviors cascade to other trophic levels. The trophic cascades are often context dependent because the same ungulates may use differing methods to avoid different predators, different ungulates may use different strategies to avoid the same predator, and different environmental context may make the same ungulate use differing methods to avoid the same predator (see previous section on trophic cascades). Moreover, strategies of using humans to reestablish the landscape of fear may only work after a lag time for the ungulate to establish that the predator is indeed a threat (Le Saout et al., 2014). Additionally, they may have limited effectiveness (or not work at all) if anthropogenic stimuli cause mismatched perception and behavioral responses in the targeted animals (Smith et al., 2021). It is this context dependency that may make using a top‐down approach difficult to apply, particularly as ecological objectives become narrower.

#### 3.4.2 Bottom‐up approaches

Few studies have directly measured the potential to apply a bottom‐up approach of managing ungulates with fear. However, several examples exist from other taxa. For example, Fernández‐Juricic et al. (2001) suggested that understanding animal responses to humans could aid in the design of parks to decrease stress‐related fear from human activity. Alternatively, that same concept could be used to cause animals to behaviorally avoid sensitive areas. For example, Blackwell et al. (2013) proposed a framework to reduce avian collisions with aircrafts by utilizing concepts in the ecology of fear to guide habitat management surrounding landing strips on airports. Clearly, vegetation structure and composition and the distribution of cover and foods also affect ungulate behavior at least in part because those factors affect predation risk. Because land management practices can drastically alter the landscape characteristics associated with vegetation, using management practices to augment trophic cascades for purposes of restoration may be possible. In support of this notion, Hebblewhite et al. (2009) reported that logging in combination with fire increased the amount of forage biomass, but elk avoided these areas because of increased predation risk from wolves in the Canadian Rockies. Thus, the dramatic change in plant community structure altered the ungulate perception of the area's riskiness, which caused them to shift behavior to avoid those areas. Similarly, Riginos and Grace (2008) reported that visual obstruction from tree density increases fear in some ungulates, which cascades to the forb community in open areas. Contrastingly, Lashley, Chitwood, Kays, et al. (2015) demonstrated that white‐tailed deer avoided areas with poor visual obstruction, even though those areas often had the greatest available nutrition (Lashley, Chitwood, Harper, et al., 2015). In all of those cases, perception of predation risk drove the animal decisions despite forage patch quality, but the antipredator behaviors of the ungulate dictated what landscape characteristics were actually avoided. Landscape structure may drive the perception of risk, meaning that manipulating landscape structure to drive a desirable trophic cascade could be possible, though many life‐history factors of the ungulate involved may confound desirable outcomes.

## 4 CONCLUSIONS

Understanding ungulate ecology of fear and its system‐wide effects would help us to better interpret ungulate ecology, improve wildlife conservation and management programs, and understand community dynamics (Teckentrup et al., 2018). Our review demonstrated that most studies of the ecology of fear can be lumped into three categories of inquiry: behavioral responses to predation risk, physiological responses to predation risk, and trophic cascades resulting from ungulate responses to predation risk. A fourth category, manipulation of predation risk, has been less studied but nonetheless represents an interesting opportunity to take research results and incorporate them into conservation and management planning (e.g., Gaynor et al., 2021). Importantly, our review suggests that collaboration across research foci (e.g., behavioral effects on physiology and how they scale to population‐level consequences) presents an opportunity to design complex research questions that have otherwise, more often than not, been treated disparately.

Our review also confirms recent work by Say‐Sallaz et al. (2019), who reported a bias in the taxa being studied and the locations in the world in which they are studied. Such bias presents a problem on multiple fronts. First, it appears that charismatic taxa and locations or events (e.g., wolf reintroduction to Yellowstone National Park) dominate the literature, meaning other predator–prey relationships and systems are not contributing proportionally to our scientific understanding of the ecology of fear. Second, many studies are limited in taxonomic scope, even when multiple predator and ungulate species are available at a given study site, which ignores the complexity associated with many predator–prey systems (Moll et al., 2017; Montgomery et al., 2019) and likely limits inference. Third, studies on movement and habitat selection dominated the topics studied under the ecology of fear paradigm, but we do not believe habitat selection alone will be enough to mechanistically explain ecology of fear. Many radiotag‐based studies are observational or opportunistic in nature. Rigorous experimental and replicated studies are required for mechanistic understanding of how fear scales to population‐level processes (see Peers et al., 2018; Prugh et al., 2019).

Ungulate responses to predation risk depend on environmental features, life‐history traits, and social structure (Ford & Goheen, 2015). However, the majority of research into “ecology of fear” focuses on elk. Additional research on less‐studied ungulates, coupled with predators with different hunting techniques, will be important to understanding the effects of fear on ungulates. We know that the two types of predation risk, individually or combined, may have different effects on ungulate responses (Creel et al., 2014, Wikenros et al., 2015, but see Dröge et al., 2017). However, the majority of research focuses on cursorial predators, and little research evaluates the effects of ambush predators or the effects of cursorial and ambush combined. Moreover, interindividual variation in traits such as boldness or shyness might play an important role affecting ungulate perception risk (Bleicher, 2017), but they are largely unstudied in the ecology of fear context.

The relationship of disease and parasitism to the ecology of fear could have important ecological, economic, or human health consequences, but the relationships between infection risk and fear responses are still largely unexplored (only 5 papers [<2%] in our review were explicitly connected to disease or parasitism). Predators may limit disease spread by reducing host densities or selecting infected individuals (Packer et al., 2003), but they could simultaneously increase transmission risk at lower ungulate densities if ungulates increase group size in response to predation risk. Also, given that host–parasite interactions potentially influence the prevalence of vector‐borne diseases, incorporating indirect effects of parasites on ungulate hosts could have implications on mitigation of disease risk (Allan et al., 2010). Understanding how non‐consumptive effects of parasitism affect host population dynamics and potentially cascade through food webs is important (Daversa et al., 2021). With numerous zoonotic pathogens transmitted via parasites, how they contribute to the ecology of fear could have implications for human health and economies.

Due to the lack of replication and difficulty of isolating trait‐mediated from density‐mediated factors, there is contrasting evidence regarding trait‐mediated trophic cascade effects on communities, ungulate populations, and ungulate physiology. Moreover, recent work highlighted concerns with sampling design that affected the strength of a trophic cascade in the Greater Yellowstone Ecosystem (Brice et al., 2022). Studies on trait‐mediated trophic cascades in particular suffer from the taxonomic and regional biases mentioned previously because they tend to be focused on cursorial predators in the Greater Yellowstone Ecosystem, likely due to the natural experiment provided by the reintroduction of wolves (Bleicher, 2017). Meanwhile, we know very little about trophic cascades generated by ambush predators (Moll et al., 2016; Thaker et al., 2011; Wikenros et al., 2015). Overcoming such bias should be fundamental to increasing our knowledge of trophic cascades. If the ecology of fear has broad importance in causing trophic cascades, avoiding bias should be fundamental to the study of its effects as well as its application to conservation and management. Given that all of the strategies we currently embrace to manipulate fear for conservation purposes are rooted in eliciting desirable trophic cascades, this may be the most important focal area for future research if we are to use the ecology of fear successfully in conservation.

If the ecology of fear is a valuable ecological paradigm, we must look beyond wolves and elk in North America and toward studies that embrace complexity in research design (as noted by Montgomery et al., 2019, Prugh et al., 2019, and Say‐Sallaz et al., 2019). Though results of studies highlighted herein often provide conflicting directionality or magnitude of effect, they provide valuable building blocks for improving future studies of ecology of fear in ungulates. The three predominate areas of research focus we identified overlap with one another extensively; recognizing they occur in an increasingly anthropogenic world (Berger et al., 2020) will be important to consider. Some authors have argued that given the pervasive effects of humans on earth, quantifying human disturbance is a high priority for conservation and that understanding the fitness costs of human activities (e.g., hiking, hunting) is an important area for future research despite the challenge for field studies (Ciuti et al., 2012, but see Schuttler et al., 2017). Only by embracing “messy projections” (Berger et al., 2020) will we be able to predict how fear might affect population dynamics and ecological processes across systems, accounting for multiple predators of varying sizes and hunting modes, with numerous prey options. We believe the current body of literature on ecology of fear comes up short on broadly explaining predator–prey dynamics in complex systems. However, the sheer number of papers on the topic demonstrate clear interest among ecologists, making future work on ecology of fear that much more valuable if it embraces complexity and expands beyond the few species and systems that have driven the development of the concept thus far. The areas of research focus identified in this review comprise a foundation for future research to link behavior, physiology, trophic cascades, and management all together as one, rather than thinking of each in a vacuum.

## ACKNOWLEDGEMENT

We thank the Associate Editor and two anonymous reviewers for thoughtful comments that improved the manuscript.

## CONFLICT OF INTEREST

Authors declare no conflict of interest.

## AUTHOR CONTRIBUTIONS

**M. Colter Chitwood:** Conceptualization (equal); Investigation (equal); Methodology (equal); Writing – original draft (lead). **Carolina Baruzzi:** Data curation (lead); Investigation (equal); Methodology (equal); Visualization (lead); Writing – original draft (supporting). **Marcus A. Lashley:** Conceptualization (equal); Investigation (equal); Methodology (equal); Writing – original draft (supporting).

## Supporting information

Appendix S1

## Data Availability

Data are provided as part of the manuscript and Appendix S1.
