# Supplementary material for: “Ecology of fear” in ungulates: Opportunities for improving conservation
Source: Ecol Evol. 2022 Mar 1;12(3):e8657. doi: 10.1002/ece3.8657 (PMC8888265; doi:10.1002/ece3.8657)
Supplement: Supplementary file 1 — Appendix S1 [file ECE3-12-e8657-s001.docx]

**Appendix Data Sources**

Abu Baker, M.A., Brown, J.S. (2014). Foraging and habitat use of common duikers, *Sylvicapra grimmia*, in a heterogeneous environment within the Soutpansberg, South Africa. *African Journal of Ecology* 52: 318-327.

Acebes, P., Malo, J.E., Traba, J. (2013). Trade-offs between food availability and predation risk in desert environments: the case of polygynous monomorphic guanaco (*Lama guanicoe*). *Journal of Arid Environments* 97: 136-142.

Ali, A.H., Ford, A.T., Evans, J.S., Mallon, D.P., Hayes, M.M., King, J., Amin, R., Goheen, J.R. (2017). Resource selection and landscape change reveal mechanisms suppressing population recovery for the world's most endangered antelope. *Journal of Applied Ecology* 54: 1720-1729.

Allan, B.F., Varns, T.S., Chase, J.M. (2010). Fear of parasites: lone star ticks increase giving-up densities in white-tailed deer. *Israel Journal of Ecology & Evolution* 56: 313-324.

Altendorf, K.B., Laundré, J.W., López González, C.A., Brown, J.S. (2001). Assessing effects of predation risk on foraging behavior of mule deer. *Journal of Mammalogy* 82: 430-439.

Anderson, D.P., Turner, M.G., Forester, J.D., Zhu, J., Boyce, M.S., Beyer, H., Stowell, L. (2005). Scale‐dependent summer resource selection by reintroduced elk in Wisconsin, USA. *The Journal of Wildlife Management* 69: 298-310.

Apollonio, M., Ciuti, S., Luccarini, S. (2005). Long-term influence of human presence on spatial sexual segregation in fallow deer (*Dama dama*). *Journal of Mammalogy* 86: 937-946.

Atickem, A., Loe, L.E., Stenseth, N.C. (2014). Individual heterogeneity in use of human shields by mountain nyala. *Ethology* 120: 715-725.

Averbeck, C., Plath, M., Wronski, T., Apio, A. (2012). Effect of human nuisance on the social organisation of large mammals: group sizes and compositions of seven ungulate species in Lake Mburo National Park and the adjacent Ankole Ranching Scheme. *Wildlife Biology* 18: 180-193.

Avgar, T., Baker, J.A., Brown, G.S., Hagens, J.S., Kittle, A.M., Mallon, E.E., McGreer, M.T., Mosser, A., Newmaster, S.G., Patterson, B.R., Reid, D.E. (2015). Space‐use behaviour of woodland caribou based on a cognitive movement model. *Journal of Animal Ecology* 84: pp.1059-1070.

Bacon, M.M., Boyce, M.S. (2016). Landscape of fear for naïve prey: Ungulates flee protected area to avoid a re-established predator. *Canadian Wildlife Biology and Management* 5:1-9.

Barnier, F., Valeix, M., Duncan, P., Chamaillé-Jammes, S., Barre, P., Loveridge, A.J., Macdonald, D.W., Fritz, H. (2014). Diet quality in a wild grazer declines under the threat of an ambush predator. *Proceedings of the Royal Society B: Biological Sciences* 281: p.20140446.

Barnier, F., Duncan, P., Fritz, H., Blanchard, P., Rubenstein, D.I., Pays, O. (2016). Between-gender differences in vigilance do not necessarily lead to differences in foraging-vigilance tradeoffs. *Oecologia* 181: 757-768.

Baruzzi, C., Lovari, S., Fattorini, N. (2017). Catch me if you can: antipredatory behaviour of chamois to the wolf. *Ethology Ecology & Evolution* 29 589-598.

Basille, M., Fortin, D., Dussault, C., Bastille-Rousseau, G., Ouellet, J.P., Courtois, R. (2015). Plastic response of fearful prey to the spatiotemporal dynamics of predator distribution. *Ecology* 96: 2622-2631.

Bastille‐Rousseau, G., Potts, J.R., Schaefer, J.A., Lewis, M.A., Ellington, E.H., Rayl, N.D., Mahoney, S.P., Murray, D.L. (2015). Unveiling trade‐offs in resource selection of migratory caribou using a mechanistic movement model of availability. *Ecography* 38: 1049-1059.

Benhaiem, S., Delon, M., Lourtet, B., Cargnelutti, B., Aulagnier, S., Hewison, A.M., Morellet, N., Verheyden, H. (2008). Hunting increases vigilance levels in roe deer and modifies feeding site selection. *Animal Behaviour* 76:611-618.

Benoist, S., Garel, M., Cugnasse, J.M., Blanchard, P. (2013). Human disturbances, habitat characteristics and social environment generate sex-specific responses in vigilance of Mediterranean mouflon. *PloS one* 8: e82960.

Berger, J., Stacey, P.B., Bellis, L., Johnson, M.P. (2001). A mammalian predator–prey imbalance: grizzly bear and wolf extinction affect avian neotropical migrants. *Ecological Applications* 11: 947-960.

Berger, J. (2007). Fear, human shields and the redistribution of prey and predators in protected areas. *Biology letters* 3: 620-623.

Berger, K.M., Gese, E.M., Berger, J. (2008). Indirect effects and traditional trophic cascades: a test involving wolves, coyotes, and pronghorn. *Ecology* 89: 818-828.

Bergvall, U.A., Svensson, L., Kjellander, P. (2016). Vigilance adjustments in relation to long-and short term risk in wild fallow deer (*Dama dama*). *Behavioural processes* 128: 58-63.

Beschta, R.L. (2003). Cottonwoods, elk, and wolves in the Lamar Valley of Yellowstone National Park. *Ecological Applications* 13:1295-1309.

Beschta, R.L., Ripple, W.J. (2006). River channel dynamics following extirpation of wolves in northwestern Yellowstone National Park, USA. *Earth Surface Processes and Landforms: The Journal of the British Geomorphological Research Group* 31: 1525-1539.

Beschta, R.L., Ripple, W.J. (2007). Increased willow heights along northern Yellowstone's Blacktail Deer Creek following wolf reintroduction. *Western North American Naturalist* 67: 613-617.

Beschta, R.L., Ripple, W.J. (2007). Wolves, elk, and aspen in the winter range of Jasper National Park, Canada. *Canadian Journal of Forest Research* 37: 1873-1885.

Beschta, R.L., Ripple, W.J. (2009). Large predators and trophic cascades in terrestrial ecosystems of the western United States. *Biological conservation* 142: 2401-2414.

Beschta, R.L., Ripple, W.J. (2010). Mexican wolves, elk, and aspen in Arizona: Is there a trophic cascade?. *Forest Ecology and Management* 260: 915-922.

Beschta, R.L., Ripple, W.J. (2010). Recovering riparian plant communities with wolves in northern Yellowstone, USA. *Restoration Ecology* 18: 380-389.

Beschta, R.L., Ripple, W.J. (2012). Berry-producing shrub characteristics following wolf reintroduction in Yellowstone National Park. *Forest Ecology and Management* 276: 132-138.

Beschta, R.L., Ripple, W.J. (2016). Riparian vegetation recovery in Yellowstone: the first two decades after wolf reintroduction. *Biological Conservation* 198: 93-103.

Beschta, R.L., Painter, L.E., Ripple, W.J. (2018). Trophic cascades at multiple spatial scales shape recovery of young aspen in Yellowstone. *Forest Ecology and Management* 413: 62-69.

Beschta, R.L., Ripple, W.J. (2018). Can large carnivores change streams via a trophic cascade?: Can large carnivores change streams?. *Ecohydrology* e2048.

Beyer, H.L., Merrill, E.H., Varley, N., Boyce, M.S. (2007). Willow on Yellowstone's northern range: evidence for a trophic cascade?. *Ecological Applications* 17: 1563-1571.

Biggerstaff, M.T., Lashley, M.A., Chitwood, M.C., Moorman, C.E., DePerno, C.S. (2017). Sexual segregation of forage patch use: support for the social-factors and predation hypotheses. *Behavioural processes* 136: 36-42.

Bjørneraas, K., Solberg, E.J., Herfindal, I., Van Moorter, B., Rolandsen, C.M., Tremblay, J.P., Skarpe, C., Sæther, B.E., Eriksen, R., Astrup, R. (2011). Moose *Alces alces* habitat use at multiple temporal scales in a human-altered landscape. *Wildlife Biology* 17: 44-54.

Blake, J.G., Mosquera, D., Loiselle, B.A., Romo, D., Swing, K. (2017). Effects of human traffic on use of trails by mammals in lowland forest of eastern Ecuador. *Neotropical Biodiversity* 3: 57-64.

Blanchard, P., Pays, O., Fritz, H. (2017). Ticks or lions: trading between allogrooming and vigilance in maternal care. *Animal Behaviour* 129: 269-279.

Blank, D.A. (2018). Vigilance, staring and escape running in antipredator behavior of goitered gazelle. *Behavioural processes* 157: 408-416.

Bonnot, N., Morellet, N., Verheyden, H., Cargnelutti, B., Lourtet, B., Klein, F., Hewison, A.M. (2013). Habitat use under predation risk: hunting, roads and human dwellings influence the spatial behaviour of roe deer. *European Journal of Wildlife Research* 59: 185-193.

Bonnot, N., Verheyden, H., Blanchard, P., Cote, J., Debeffe, L., Cargnelutti, B., Klein, F., Hewison, M.A.J., Morellet, N. (2014). Interindividual variability in habitat use: evidence for a risk management syndrome in roe deer?. *Behavioral Ecology* 26: 105-114.

Boulanger, J., Poole, K.G., Gunn, A., Wierzchowski, J. (2012). Estimating the zone of influence of industrial developments on wildlife: a migratory caribou Rangifer tarandus groenlandicus and diamond mine case study. *Wildlife Biology* 18: 164-179.

Bourbeau‐Lemieux, A., Festa‐Bianchet, M., Gaillard, J.M., Pelletier, F. (2011). Predator‐driven component Allee effects in a wild ungulate. *Ecology Letters* 14: 358-363.

Bouyer, Y., Rigot, T., Panzacchi, M., Van Moorter, B., Poncin, P., Beudels-Jamar, R., Odden, J., Linnell, J.D. (2015). Using zero-inflated models to predict the relative distribution and abundance of roe deer over very large spatial scales. In *Annales Zoologici Fennici* 52: 66-76. Finnish Zoological and Botanical Publishing.

Brambilla, A., von Hardenberg, A., Kristo, O., Bassano, B., Bogliani, G. (2013). Don't spit in the soup: faecal avoidance in foraging wild Alpine ibex, *Capra ibex*. *Animal Behaviour* 86: 153-158.

Brivio, F., Grignolio, S., Brambilla, A., Apollonio, M. (2014). Intra-sexual variability in feeding behaviour of a mountain ungulate: size matters. *Behavioral ecology and sociobiology* 68: 1649-1660.

Brown, C.L., Hardy, A.R., Barber, J.R., Fristrup, K.M., Crooks, K.R., Angeloni, L.M. (2012). The effect of human activities and their associated noise on ungulate behavior. *PloS one* 7: e40505.

Burkepile, D.E., Burns, C.E., Tambling, C.J., Amendola, E., Buis, G.M., Govender, N., Nelson, V., Thompson, D.I., Zinn, A.D., Smith, M.D. (2013). Habitat selection by large herbivores in a southern African savanna: the relative roles of bottom-up and top-down forces. *Ecosphere* 4: 139.

Cappa, F., Campos, V., Giannoni, S., Andino, N. (2017). The effects of poaching and habitat structure on anti-predator behavioral strategies: A guanaco population in a high cold desert as case study. *PloS one* 12: e0184018.

Cappa, F.M., Giannoni, S.M., Borghi, C.E. (2017). Effects of roads on the behaviour of the largest South American artiodactyl (*Lama guanicoe*) in an Argentine reserve. *Animal Behaviour* 131: 131-136.

Chamaillé-Jammes, S., Malcuit, H., Le Saout, S., Martin, J.L. (2014). Innate threat-sensitive foraging: black-tailed deer remain more fearful of wolf than of the less dangerous black bear even after 100 years of wolf absence. *Oecologia* 174: 1151-1158.

Cherry, M.J., Conner, L.M., Warren, R.J. (2015). Effects of predation risk and group dynamics on white-tailed deer foraging behavior in a longleaf pine savanna. *Behavioral Ecology* 26: 1091-1099.

Cherry, M.J., Morgan, K.E., Rutledge, B.T., Conner, L.M., Warren, R.J. (2016). Can coyote predation risk induce reproduction suppression in white‐tailed deer?. *Ecosphere* 7: e01481.

Cherry, M.J., Warren, R.J., Conner, L.M. (2016). Fear, fire, and behaviorally mediated trophic cascades in a frequently burned savanna. *Forest Ecology and Management* 368: 133-139.

Childress, M.J., Lung, M.A. (2003). Predation risk, gender and the group size effect: does elk vigilance depend upon the behaviour of conspecifics?. *Animal Behaviour* 66: 389-398.

Christianson, D., Scott C. (2010). A nutritionally mediated risk effect of wolves on elk. *Ecology* 91: 1184-1191.

Christianson, D., Creel, S. (2014). Ecosystem scale declines in elk recruitment and population growth with wolf colonization: a before-after-control-impact approach. *PloS one* 9: p.e102330.

Christianson, D., Becker, M.S., Brennan, A., Creel, S., Dröge, E., M'soka, J., Mukula, T., Schuette, P., Smit, D., Watson, F. (2018). Foraging investment in a long‐lived herbivore and vulnerability to coursing and stalking predators. *Ecology and Evolution* 8: 10147-10155.

Ciach, M., Pęksa, Ł. (2018). Human-induced environmental changes influence habitat use by an ungulate over the long term. *Current Zoology* 65: 129-137.

Ciuti, S., Muhly, T.B., Paton, D.G., McDevitt, A.D., Musiani, M., Boyce, M.S. (2012). Human selection of elk behavioural traits in a landscape of fear. *Proceedings of the Royal Society of London B: Biological Sciences* rspb20121483.

Ciuti, S., Northrup, J.M., Muhly, T.B., Simi, S., Musiani, M., Pitt, J.A., Boyce, M.S. (2012). Effects of humans on behaviour of wildlife exceed those of natural predators in a landscape of fear. *PloS one* 7: e50611.

Cooke, R.S., Woodfine, T., Petretto, M., Ezard, T.H. (2016). Resource partitioning between ungulate populations in arid environments. *Ecology and Evolution* 6: 6354-6365.

Coppes, J., Burghardt, F., Hagen, R., Suchant, R., Braunisch, V. (2017). Human recreation affects spatio-temporal habitat use patterns in red deer (*Cervus elaphus*). *PloS one* 12: e0175134.

Coulon, A., Morellet, N., Goulard, M., Cargnelutti, B., Angibault, J.M., Hewison, A.M. (2008). Inferring the effects of landscape structure on roe deer (*Capreolus capreolus*) movements using a step selection function. *Landscape Ecology* 23: 603-614.

Courbin, N., Loveridge, A., Fritz, H., Macdonald, D., Patin, R., Valeix, M., Chamaille-Jammes, S. (2017). Zebra diel migrations reduce encounter risk with lions over selection for safe habitats. *bioRxiv* 165597.

Creel, S., Winnie Jr, J., Maxwell, B., Hamlin, K., Creel, M. (2005). Elk alter habitat selection as an antipredator response to wolves. *Ecology* 86: 3387-3397.

Creel, S., Winnie Jr, J.A., Christianson, D., Liley, S. (2008). Time and space in general models of antipredator response: tests with wolves and elk. *Animal Behaviour* 76: 1139-1146.

Creel, S., Winnie, J.A., Christianson, D. (2009). Glucocorticoid stress hormones and the effect of predation risk on elk reproduction. *Proceedings of the National Academy of Sciences* 106: 12388-12393.

Creel, S., Christianson, D. (2009). Wolf presence and increased willow consumption by Yellowstone elk: implications for trophic cascades. *Ecology* 90: 2454-2466.

Creel, S., Schuette, P., Christianson, D. (2014). Effects of predation risk on group size, vigilance, and foraging behavior in an African ungulate community. *Behavioral Ecology* 25: 773-784.

Crosmary, W.G., Valeix, M., Fritz, H., Madzikanda, H., Côté, S.D. (2012). African ungulates and their drinking problems: hunting and predation risks constrain access to water. *Animal Behaviour* 83: 145-153.

Dalerum, F., Belton, L. (2014). African ungulates recognize a locally extinct native predator. *Behavioral Ecology* 26: 215-222.

Davies, A.B., Tambling, C.J., Kerley, G.I., Asner, G.P. (2016). Limited spatial response to direct predation risk by African herbivores following predator reintroduction. *Ecology and Evolution* 6: 5728-5748.

DeMars, C.A., Boutin, S. (2018). Nowhere to hide: Effects of linear features on predator–prey dynamics in a large mammal system. *Journal of Animal Ecology* 87: 274-284.

Ditmer, M.A., Fieberg, J.R., Moen, R.A., Windels, S.K., Stapleton, S.P., Harris, T.R. (2018). Moose movement rates are altered by wolf presence in two ecosystems. *Ecology and Evolution* 8: 9017-9033.

Djagoun, C.A., Kassa, B., Djossa, B.A., Coulson, T., Mensah, G.A., Sinsin, B. (2014). Hunting affects dry season habitat selection by several bovid species in northern Benin. *Wildlife Biology* 20: 83-90.

Dröge, E., Creel, S., Becker, M.S., M’soka, J. (2017). Risky times and risky places interact to affect prey behaviour. *Nature Ecology & Evolution* 1: 1123.

Drolet, A., Dussault, C., Côté, S.D. (2016). Simulated drilling noise affects the space use of a large terrestrial mammal. *Wildlife Biology* 22: 284-293.

Duquette, J.F., Belant, J.L., Svoboda, N.J., Beyer Jr, D.E., Lederle, P.E. (2015). Scale Dependence of Female Ungulate Reproductive Success in Relation to Nutritional Condition, Resource Selection and Multi-Predator Avoidance. *PloS one* 10: e0140433.

Dwinnell, S.P., Sawyer, H., Randall, J.E., Beck, J.L., Forbey, J.S., Fralick, G.L., Monteith, K.L. (2019). Where to forage when afraid: Does perceived risk impair use of the foodscape?. *Ecological Applications* e01972.

Dzialak, M.R., Webb, S.L., Harju, S.M., Winstead, J.B., Wondzell, J.J., Mudd, J.P., Hayden-Wing, L.D. (2011). The spatial pattern of demographic performance as a component of sustainable landscape management and planning. *Landscape Ecology* 26: 775-790.

Eby, S.L., Anderson, T.M., Mayemba, E.P., Ritchie, M.E. (2014). The effect of fire on habitat selection of mammalian herbivores: the role of body size and vegetation characteristics. *Journal of Animal Ecology* 83: 1196-1205.

Eccard, J.A., Meißner, J.K., Heurich, M. (2017). European roe deer increase vigilance when faced with immediate predation risk by Eurasian Lynx. *Ethology* 123: 30-40.

Eisenberg, C., Hibbs, D.E., Ripple, W.J., Salwasser, H. (2014). Context dependence of elk (*Cervus elaphus*) vigilance and wolf (*Canis lupus*) predation risk. *Canadian Journal of Zoology* 92: 727-736.

Eisenberg, C., Hibbs, D.E., Ripple, W.J. (2015). Effects of predation risk on elk (Cervus elaphus) landscape use in a wolf (Canis lupus) dominated system. *Canadian Journal of Zoology* 93: 99-111.

Esparza-Carlos, J.P., Laundré, J.W., Hernández, L., Íñiguez-Dávalos, L.I. (2016). Apprehension affecting foraging patterns and landscape use of mule deer in arid environments. *Mammalian Biology-Zeitschrift für Säugetierkunde* 81: 543-550.

Ezenwa, V.O. (2004). Selective defecation and selective foraging: antiparasite behavior in wild ungulates?. *Ethology* 110: 851-862.

Fattebert, J., Baubet, E., Slotow, R., Fischer, C. (2017). Landscape effects on wild boar home range size under contrasting harvest regimes in a human-dominated agro-ecosystem. *European Journal of Wildlife Research* 63: 32.

Flagel, D.G., Belovsky, G.E., Beyer, D.E. (2016). Natural and experimental tests of trophic cascades: gray wolves and white-tailed deer in a Great Lakes forest. *Oecologia* 180: 1183-1194.

Ford, A.T., Goheen, J.R., Otieno, T.O., Bidner, L., Isbell, L.A., Palmer, T.M., Ward, D., Woodroffe, R., Pringle, R.M. (2014). Large carnivores make savanna tree communities less thorny. *Science* 346: 346-349.

Ford, A.T., Goheen, J.R., Augustine, D.J., Kinnaird, M.F., O'Brien, T.G., Palmer, T.M., Pringle, R.M., Woodroffe, R. (2015). Recovery of African wild dogs suppresses prey but does not trigger a trophic cascade. *Ecology* 96: 2705-2714.

Ford, A.T., Goheen, J.R. (2015). An experimental study on risk effects in a dwarf antelope, *Madoqua guentheri*. *Journal of Mammalogy* 96: 918-926.

Fortin, D., Beyer, H.L., Boyce, M.S., Smith, D.W., Duchesne, T., Mao, J.S. (2005). Wolves influence elk movements: behavior shapes a trophic cascade in Yellowstone National Park. *Ecology* 86: 1320-1330.

Fortin, D., Fortin, M.E. (2009). Group-size-dependent association between food profitability, predation risk and distribution of free-ranging bison. *Animal Behaviour* 78: 887-892.

Found, R., Clair, C.S. (2016). Behavioural syndromes predict loss of migration in wild elk. *Animal Behaviour* 115: 35-46.

Frank, D.A. (2008). Evidence for top predator control of a grazing ecosystem. *Oikos* 117: 1718-1724.

Frank, D.A., Wallen, R.L., White, P.J. (2016). Ungulate control of grassland production: grazing intensity and ungulate species composition in Yellowstone Park. *Ecosphere* 7: e01603.

Gehr, B., Hofer, E.J., Pewsner, M., Ryser, A., Vimercati, E., Vogt, K., Keller, L.F. (2018). Hunting‐mediated predator facilitation and superadditive mortality in a European ungulate. *Ecology and Evolution* 8: 109-119.

Gehr, B., Hofer, E.J., Ryser, A., Vimercati, E., Vogt, K., Keller, L.F. (2018). Evidence for nonconsumptive effects from a large predator in an ungulate prey?. *Behavioral Ecology* 29: 724-735.

Gray, E.F., Bond, W.J. (2013). Will woody plant encroachment impact the visitor experience and economy of conservation areas?. *Koedoe* 55: 00-00.

Gregory, A.J., Lung, M.A., Gehring, T.M., Swanson, B.J. (2009). The importance of sex and spatial scale when evaluating sexual segregation by elk in Yellowstone. *Journal of Mammalogy* 90: 971-979.

Grignolio, S., Merli, E., Bongi, P., Ciuti, S., Apollonio, M. (2011). Effects of hunting with hounds on a non-target species living on the edge of a protected area. *Biological Conservation* 144: 641-649.

Gower, C.N., Garrott, R.A., White, P.J., Cherry, S., Yoccoz, N.G. (2008). Elk group size and wolf predation: a flexible strategy when faced with variable risk. *Terrestrial Ecology* 3: 401-422.

Gude, J.A., Garrott, R.A., Borkowski, J.J., King, F. (2006). Prey risk allocation in a grazing ecosystem. *Ecological Applications* 16: 285-298.

Gulsby, W.D., Cherry, M.J., Johnson, J.T., Conner, L.M., Miller, K.V. (2018). Behavioral response of white‐tailed deer to coyote predation risk. *Ecosphere* 9: e02141.

Halofsky, J., Ripple, W. (2008). Linkages between wolf presence and aspen recruitment in the Gallatin elk winter range of southwestern Montana, USA. *Forestry* 81: 195-207.

Halofsky, J.S., Ripple W.J. (2008). Fine-scale predation risk on elk after wolf reintroduction in Yellowstone National Park, USA. *Oecologia* 155: 869-877.

Halofsky, J.S., Ripple, W.J., Beschta, R.L. (2008). Recoupling fire and aspen recruitment after wolf reintroduction in Yellowstone National Park, USA. *Forest Ecology and Management* 256: 1004-1008.

Hamel, S., Côté S.D. (2007). Habitat use patterns in relation to escape terrain: are alpine ungulate females trading off better foraging sites for safety?. *Canadian Journal of Zoology* 85: 933-943.

Harvey, L., Fortin, D. (2013). Spatial heterogeneity in the strength of plant-herbivore interactions under predation risk: the tale of bison foraging in wolf country. *PloS one* 8: e73324.

Hebblewhite, M., Pletscher, D.H. (2002). Effects of elk group size on predation by wolves. *Canadian Journal of Zoology* 80: 800-809.

Hebblewhite, M., White, C.A., Nietvelt, C.G., McKenzie, J.A., Hurd, T.E., Fryxell, J.M., Bayley, S.E., Paquet, P.C. (2005). Human activity mediates a trophic cascade caused by wolves. *Ecology* 86: 2135-2144.

Hebblewhite, M., Merrill E.H. (2009). Trade‐offs between predation risk and forage differ between migrant strategies in a migratory ungulate. *Ecology* 90: 3445-3454.

Hernández, L., Laundré J.W. (2005). Foraging in the ‘landscape of fear’ and its implications for habitat use and diet quality of elk *Cervus elaphus* and bison *Bison bison*. *Wildlife Biology* 11: 215-220.

Hochman, V., Kotler, B.P. (2006). Patch use, apprehension, and vigilance behavior of Nubian Ibex under perceived risk of predation. *Behavioral Ecology* 18: 368-374.

Hollenbeck, J.P., Ripple, W.J. (2008). Aspen snag dynamics, cavity-nesting birds, and trophic cascades in Yellowstone's northern range. *Forest Ecology and Management* 255: 1095-1103.

Hopcraft, J.G.C., Anderson, T.M., Pérez‐Vila, S., Mayemba, E., Olff, H. (2012). Body size and the division of niche space: food and predation differentially shape the distribution of Serengeti grazers. *Journal of Animal Ecology* 81: 201-213.

Howe, M., Okello, M.M., Davis, J.M. (2013). Interspecific variation in the distribution of ungulates relative to human infrastructure surrounding Amboseli National Park. *African Zoology* 48: 159-166.

Iranzo, E.C., Wittmer, H.U., Traba, J., Acebes, P., Mata, C., Malo, J.E. (2018). Predator occurrence and perceived predation risk determine grouping behavior in guanaco (*Lama guanicoe*). *Ethology* 124: 281-289.

Iribarren, C., Kotler, B.P. (2012). Foraging patterns of habitat use reveal landscape of fear of Nubian ibex *Capra nubiana*. *Wildlife Biology* 18: 194-201.

Jarnemo, A., Wikenros, C. (2014). Movement pattern of red deer during drive hunts in Sweden. *European Journal of Wildlife Research* 60: 77-84.

Jayakody, S., Sibbald, A.M., Gordon, I.J., Lambin, X. (2008). Red deer *Cervus elephus* vigilance behaviour differs with habitat and type of human disturbance. *Wildlife Biology* 14: 81-91.

Jiang, G., Zhang, M., Ma, J. (2007). Effects of human disturbance on movement, foraging and bed selection in red deer *Cervus elaphus xanthopygus* from the Wandashan Mountains, northeastern China. *Acta Theriologica* 52: 435-446.

Jiang, G., Zhang, M., Ma, J. (2008). Habitat use and separation between red deer *Cervus elaphus xanthopygus* and roe deer *Capreolus pygargus bedfordi* in relation to human disturbance in the Wandashan Mountains, northeastern China. *Wildlife Biology* 14: 92-100.

Johnston, D.B., Cooper, D.J., Hobbs, N.T. (2007). Elk browsing increases aboveground growth of water-stressed willows by modifying plant architecture. *Oecologia* 154: 467-478.

Kauffman, M.J., Brodie, J.F., Jules, E.S. (2010). Are wolves saving Yellowstone's aspen? A landscape‐level test of a behaviorally mediated trophic cascade. *Ecology* 91: 2742-2755.

Kay, C.E. (2001). Long-term aspen exclosures in the Yellowstone ecosystem. In *In: Shepperd, Wayne D.; Binkley, Dan; Bartos, Dale L.; Stohlgren, Thomas J.; Eskew, Lane G., comps. Sustaining aspen in western landscapes: Symposium proceedings; 13-15 June 2000; Grand Junction, CO. Proceedings RMRS-P-18. Fort Collins, CO: US Department of Agriculture, Forest Service, Rocky Mountain Research Station* 18: 225-242.

Kimble, D.S., Tyers, D.B., Sowell, B.F. (2011). Quaking aspen ecology on forest service lands north of Yellowstone National Park. *Natural Resources and Environmental Issues* 16: 8.

Kimble, D.S., Tyers, D.B., Robison-Cox, J., Sowell, B.F. (2011). Aspen recovery since wolf reintroduction on the northern Yellowstone winter range. *Rangeland Ecology & Management* 64: 119-130.

Kitchen, D.M., Bergman, T.J., Cheney, D.L., Nicholson, J.R., Seyfarth, R.M. (2010). Comparing responses of four ungulate species to playbacks of baboon alarm calls. *Animal Cognition* 13: 861-870.

Kittle, A.M., Fryxell, J.M., Desy, G.E., Hamr, J. (2008). The scale-dependent impact of wolf predation risk on resource selection by three sympatric ungulates. *Oecologia* 157: 163-175.

Kohl, M.T., Stahler, D.R., Metz, M.C., Forester, J.D., Kauffman, M.J., Varley, N., White, P.J., Smith, D.W., MacNulty, D.R. (2018). Diel predator activity drives a dynamic landscape of fear. *Ecological Monographs* 88: 638-652.

Krishna, Y.C., Kumar, A., Isvaran, K. (2016). Wild ungulate decision-Making and the role of tiny refuges in human-dominated landscapes. *PloS one* 11: e0151748.

Kuijper, D.P.J., De Kleine, C., Churski, M., Van Hooft, P., Bubnicki, J., Jędrzejewska, B. (2013). Landscape of fear in Europe: wolves affect spatial patterns of ungulate browsing in Białowieża Primeval Forest, Poland. *Ecography* 36: 1263-1275.

Kuijper, D.P., Verwijmeren, M., Churski, M., Zbyryt, A., Schmidt, K., Jędrzejewska, B., Smit, C. (2014). What cues do ungulates use to assess predation risk in dense temperate forests?. *PLoS One* 9: p.e84607.

Kuijper, D.P., Bubnicki, J.W., Churski, M., Mols, B., Van Hooft, P. (2015). Context dependence of risk effects: wolves and tree logs create patches of fear in an old-growth forest. *Behavioral Ecology* 26: 1558-1568.

Lashley, M.A., Chitwood, M.C., Biggerstaff, M.T., Morina, D.L., Moorman, C.E., DePerno, C.S. (2014). White-tailed deer vigilance: the influence of social and environmental factors. *PLoS One* 9: e90652.

Laundré, J.W., Hernández, L., Altendorf, K.B. (2001). Wolves, elk, and bison: reestablishing the "landscape of fear" in Yellowstone National Park, USA. *Canadian Journal of Zoology* 79: 1401-1409.

Laundré, J.W. (2010). Behavioral response races, predator–prey shell games, ecology of fear, and patch use of pumas and their ungulate prey. *Ecology* 91: 2995-3007.

Laporte, I., Muhly, T.B., Pitt, J.A., Alexander, M., Musiani, M. (2010). Effects of wolves on elk and cattle behaviors: implications for livestock production and wolf conservation. *PloS one* 5: e11954.

Leblond, M., Dussault, C., Ouellet, J.P. (2013). Impacts of human disturbance on large prey species: do behavioral reactions translate to fitness consequences?. *PLoS One* 8: e73695.

Leblond, M., Dussault, C., Ouellet, J.P., St‐Laurent, M.H. (2016). Caribou avoiding wolves face increased predation by bears–Caught between Scylla and Charybdis. *Journal of Applied Ecology* 53: 1078-1087.

Le Roux, E., Kerley, G.I., Cromsigt, J.P. (2018). Megaherbivores modify trophic cascades triggered by fear of predation in an African savanna ecosystem. *Current Biology* 28: 2493-2499.

Le Saout, S., Padié, S., Chamaillé-Jammes, S., Chollet, S., Côté, S., Morellet, N., Pattison, J., Harris, E., Martin, J.L. (2014). Short-term effects of hunting on naïve black-tailed deer (*Odocoileus hemionus sitkensis*): behavioural response and consequences on vegetation growth. *Canadian Journal of Zoology* 92: 915-925.

Le Saout, S., Martin, J.L., Blanchard, P., Cebe, N., Mark Hewison, A.J., Rames, J.L., Chamaillé‐Jammes, S. (2015). Seeing a ghost? Vigilance and its drivers in a predator‐free world. *Ethology* 121: 651-660.

Le Saout, S., Massouh, M., Martin, J.L., Presseault-Gauvin, H., Poilvé, E., Côté, S.D., Picot, D., Verheyden, H., Chamaillé-Jammes, S. (2016). Levels of fecal glucocorticoid metabolites do not reflect environmental contrasts across islands in black-tailed deer (*Odocoileus hemionus sitkensis*) populations. *Mammal Research* 61: 391-398.

Lesmerises, F., Johnson, C.J., St‐Laurent, M.H. (2017). Refuge or predation risk? Alternate ways to perceive hiker disturbance based on maternal state of female caribou. *Ecology and Evolution* 7: 845-854.

Lesmerises, F., Déry, F., Johnson, C.J., St-Laurent, M.H. (2018). Spatiotemporal response of mountain caribou to the intensity of backcountry skiing. *Biological Conservation* 217: 149-156.

Lone, K., Loe, L.E., Gobakken, T., Linnell, J.D., Odden, J., Remmen, J., Mysterud, A. (2014). Living and dying in a multi‐predator landscape of fear: roe deer are squeezed by contrasting pattern of predation risk imposed by lynx and humans. *Oikos* 123: 641-651.

Lone, K., Loe, L.E., Meisingset, E.L., Stamnes, I., Mysterud, A. (2015). An adaptive behavioural response to hunting: surviving male red deer shift habitat at the onset of the hunting season. *Animal Behaviour* 102: 127-138.

Losier, C.L., Couturier, S., St‐Laurent, M.H., Drapeau, P., Dussault, C., Rudolph, T., Brodeur, V., Merkle, J.A., Fortin, D. (2015). Adjustments in habitat selection to changing availability induce fitness costs for a threatened ungulate. *Journal of Applied Ecology* 52: 496-504.

Lung, M.A., Childress, M.J. (2006). The influence of conspecifics and predation risk on the vigilance of elk (*Cervus elaphus*) in Yellowstone National Park. *Behavioral Ecology* 18: 12-20.

Luo, Z., Liu, B., Liu, S., Jiang, Z., Halbrook, R.S. (2014). Influences of human and livestock density on winter habitat selection of Mongolian gazelle (*Procapra gutturosa*). *Zoological Science* 31: 20-30.

Lynch, E., Northrup, J.M., McKenna, M.F., Anderson Jr, C.R., Angeloni, L., Wittemyer, G. (2014). Landscape and anthropogenic features influence the use of auditory vigilance by mule deer. *Behavioral Ecology* 26: 75-82.

Magle, S.B., Simoni, L.S., Lehrer, E.W., Brown, J.S. (2014). Urban predator–prey association: coyote and deer distributions in the Chicago metropolitan area. *Urban Ecosystems* 17: 875-891.

Makin, D.F., Chamaillé-Jammes, S., Shrader, A.M. (2017). Changes in feeding behavior and patch use by herbivores in response to the introduction of a new predator. *Journal of Mammalogy* 99: 341-350.

Makin, D.F., Chamaillé-Jammes, S., Shrader, A.M. (2017). Herbivores employ a suite of antipredator behaviours to minimize risk from ambush and cursorial predators. *Animal Behaviour* 127: 225-231.

Månsson, J., Prima, M.C., Nicholson, K.L., Wikenros, C., Sand, H. (2017). Group or ungroup–moose behavioural response to recolonization of wolves. *Frontiers in Zoology* 14: 10.

Mao, J.S., Boyce, M.S., Smith, D.W., Singer, F.J., Vales, D.J., Vore, J.M., Merrill, E.H. (2005). Habitat selection by elk before and after wolf reintroduction in Yellowstone National Park. *The Journal of Wildlife Management* 69: 1691-1707.

Marantz, S.A., Long, J.A., Webb, S.L., Gee, K.L., Little, A.R., Demarais, S. (2016). Impacts of human hunting on spatial behavior of white-tailed deer (*Odocoileus virginianus*). *Canadian Journal of Zoology* 94: 853-861.

Marchand, P., Garel, M., Bourgoin, G., Dubray, D., Maillard, D., Loison, A. (2014). Impacts of tourism and hunting on a large herbivore’s spatio-temporal behavior in and around a French protected area. *Biological Conservation* 177: 1-11.

Marie, M.K., Adam, W., Zbigniew, B. (2018). Effects of forest roads on oak trees via cervid habitat use and browsing. *Forest Ecology and Management* 424: 378-386.

Marshall, K.N., Hobbs, N.T., Cooper, D.J. (2013). Stream hydrology limits recovery of riparian ecosystems after wolf reintroduction. *Proceedings of the Royal Society of London B: Biological Sciences* 280: 20122977.

Marshall, K.N., Cooper, D.J., Hobbs, N.T. (2014). Interactions among herbivory, climate, topography and plant age shape riparian willow dynamics in northern Yellowstone National Park, USA. *Journal of Ecology* 102: 667-677.

Martin, J., Owen-Smith, N. (2016). Habitat selectivity influences the reactive responses of African ungulates to encounters with lions. *Animal Behaviour* 116: 163-170.

Martin, J., Vourc’h, G., Bonnot, N., Cargnelutti, B., Chaval, Y., Lourtet, B., Goulard, M., Hoch, T., Plantard, O., Hewison, A.M., Morellet, N. (2018). Temporal shifts in landscape connectivity for an ecosystem engineer, the roe deer, across a multiple-use landscape. *Landscape Ecology* 33: 937-954.

Middleton, A.D., Kauffman, M.J., McWhirter, D.E., Jimenez, M.D., Cook, R.C., Cook, J.G., Albeke, S.E., Sawyer, H., White, P.J. (2013). Linking anti‐predator behaviour to prey demography reveals limited risk effects of an actively hunting large carnivore. *Ecology Letters* 16: 1023-1030.

Moll, R.J., Killion, A.K., Montgomery, R.A., Tambling, C.J., Hayward, M.W. (2016). Spatial patterns of African ungulate aggregation reveal complex but limited risk effects from reintroduced carnivores. *Ecology* 97: 1123-1134. doi: 10.1890/15-0707.1

Montgomery, R.A., Roloff, G.J., Millspaugh, J.J. (2012). Importance of visibility when evaluating animal response to roads. *Wildlife Biology* 18: 393-405. doi:

Montgomery, R.A., Vucetich, J.A., Peterson, R.O., Roloff, G.J., Millenbah, K.F. (2013). The influence of winter severity, predation and senescence on moose habitat use. *Journal of Animal Ecology* 82: 301-309. doi:

Mooring, M.S., Fitzpatrick, T.A., Nishihira, T.T., Reisig, D.D. (2004). Vigilance, predation risk, and the Allee effect in desert bighorn sheep. *The Journal of Wildlife Management* 68: 519-532. doi:

M'soka, J., Creel, S., Becker, M.S., Murdoch, J.D. (2017). Ecological and anthropogenic effects on the density of migratory and resident ungulates in a human‐inhabited protected area. *African Journal of Ecology* 55: 618-631. doi:

Muhly, T.B., Semeniuk, C., Massolo, A., Hickman, L., Musiani, M. (2011). Human activity helps prey win the predator-prey space race. *PLoS One* 6: e17050. doi:

Mulero‐Pázmány, M., D'Amico, M., González‐Suárez, M. (2016). Ungulate behavioral responses to the heterogeneous road‐network of a touristic protected area in Africa. *Journal of Zoology* 298: 233-240. doi:

Müller, A., Dahm, M., Bøcher, P.K., Root-Bernstein, M., Svenning, J.C. (2017). Large herbivores in novel ecosystems-Habitat selection by red deer (*Cervus elaphus*) in a former brown-coal mining area. *PloS one* 12: e0177431. doi:

Mumma, M.A., Gillingham, M.P., Johnson, C.J., Parker, K.L. (2017). Understanding predation risk and individual variation in risk avoidance for threatened boreal caribou. *Ecology and Evolution* 7: 10266-10277. doi:

Muposhi, V.K., Gandiwa, E., Chemura, A., Bartels, P., Makuza, S.M., Madiri, T.H. (2016). Habitat heterogeneity variably influences habitat selection by wild herbivores in a semi-arid tropical savanna ecosystem. *PloS one* 11: e0163084. doi:

Neumann, W., Ericsson, G., Dettki, H. (2009). The non-impact of hunting on moose *Alces alces* movement, diurnal activity, and activity range. *European Journal of Wildlife Research* 55: 255-265. doi:

Neumann, W., Ericsson, G., Dettki, H. (2010). Does off-trail backcountry skiing disturb moose?. *European Journal of Wildlife Research* 56: 513-518. doi:

Ng’weno, C.C., Maiyo, N.J., Ali, A.H., Kibungei, A.K., Goheen, J.R. (2017). Lions influence the decline and habitat shift of hartebeest in a semiarid savanna. *Journal of Mammalogy* 98: 1078-1087. doi:

Nicholson, K.L., Milleret, C., Månsson, J., Sand, H. (2014). Testing the risk of predation hypothesis: the influence of recolonizing wolves on habitat use by moose. *Oecologia* 176: 69-80. doi:

Nikula, A., Heikkinen, S., Helle, E. (2004). Habitat selection of adult moose *Alces alces* at two spatial scales in central Finland. *Wildlife Biology* 10: 121-135. doi:

Nuttle, T., Yerger, E.H., Stoleson, S.H., Ristau, T.E. (2011). Legacy of top‐down herbivore pressure ricochets back up multiple trophic levels in forest canopies over 30 years. *Ecosphere* 2: 1-11. doi:

O’Brien, P.P., Webber, Q.M., Vander Wal, E. (2018). Consistent individual differences and population plasticity in network-derived sociality: An experimental manipulation of density in a gregarious ungulate. *PloS one* 13: e0193425. doi:

Oehlers, S.A., Bowyer, R.T., Huettmann, F., Person, D.K., Kessler, W.B. (2011). Sex and scale: implications for habitat selection by Alaskan moose *Alces alces gigas*. *Wildlife Biology* 17: 67-84. doi:

Ohashi, H., Saito, M., Horie, R., Tsunoda, H., Noba, H., Ishii, H., Kuwabara, T., Hiroshige, Y., Koike, S., Hoshino, Y., Toda, H. (2013). Differences in the activity pattern of the wild boar *Sus scrofa* related to human disturbance. *European Journal of Wildlife Research* 59: 167-177. doi:

Osada, K., Miyazono, S., Kashiwayanagi, M. (2014). Pyrazine analogs are active components of wolf urine that induce avoidance and fear-related behaviors in deer. *Frontiers in Behavioral Neuroscience* 8: 276. doi:

Owen‐Smith, N., Traill, L.W. (2017). Space use patterns of a large mammalian herbivore distinguished by activity state: fear versus food?. *Journal of Zoology* 303: 281-290. doi:

Padié, S., Morellet, N., Hewison, A.M., Martin, J.L., Bonnot, N., Cargnelutti, B., Chamaillé‐Jammes, S. (2015). Roe deer at risk: teasing apart habitat selection and landscape constraints in risk exposure at multiple scales. *Oikos* 124: 1536-1546. doi:

Painter, L.E., Ripple, W.J. (2012). Effects of bison on willow and cottonwood in northern Yellowstone National Park. *Forest Ecology and Management* 264: 150-158. doi:

Painter, L.E., Beschta, R.L., Larsen, E.J., Ripple, W.J. (2014). After long-term decline, are aspen recovering in northern Yellowstone?. *Forest Ecology and Management* 329: 108-117. doi:

Palmer, M.S., Fieberg, J., Swanson, A., Kosmala, M., Packer, C. (2017). A ‘dynamic’landscape of fear: Prey responses to spatiotemporal variations in predation risk across the lunar cycle. *Ecology Letters* 20: 1364-1373. doi:

Pan, D., Song, Y.L., Zeng, Z.G., Bravery, B.D. (2014). Habitat Selection by Eld’s Deer following Relocation to a Patchy Landscape. *PloS one* 9: e91158. doi:

Paton, D.G., Ciuti, S., Quinn, M., Boyce, M.S. (2017). Hunting exacerbates the response to human disturbance in large herbivores while migrating through a road network. *Ecosphere* 8: e01841. doi:

Pays, O., Blanchard, P., Valeix, M., Chamaillé-Jammes, S., Duncan, P., Périquet, S., Lombard, M., Ncube, G., Tarakini, T., Makuwe, E., Fritz, H. (2012). Detecting predators and locating competitors while foraging: an experimental study of a medium-sized herbivore in an African savanna. *Oecologia* 169: 419-430. doi:

Pecorella, I., Ferretti, F., Sforzi, A., Macchi, E. (2016). Effects of culling on vigilance behaviour and endogenous stress response of female fallow deer. *Wildlife Research* 43: 189-196. doi:

Pęksa, Ł., Ciach, M. (2015). Negative effects of mass tourism on high mountain fauna: the case of the Tatra chamois *Rupicapra rupicapra tatrica*. *Oryx* 49: 500-505. doi:

Périquet, S., Todd-Jones, L., Valeix, M., Stapelkamp, B., Elliot, N., Wijers, M., Pays, O., Fortin, D., Madzikanda, H., Fritz, H., Loveridge, A.J., Macdonald, D.W. (2012). Influence of immediate predation risk by lions on the vigilance of prey of different body size. *Behavioral Ecology* 23: 970-976. doi:

Périquet, S., Richardson, P., Cameron, E.Z., Ganswindt, A., Belton, L., Loubser, E., Dalerum, F. (2017). Effects of lions on behaviour and endocrine stress in plains zebras. *Ethology* 123: 667-674. doi:

Pierce, J.L., Dalinsky, S.A., Chenaille, A.A., Lolya, L.M., Maguder, J.L., Mattilio, C., Mayhew, G.W., Regan, R., Patrick, D.A. (2015). Scale-dependent effects of coyote-predation risk on patterns of white-tailed deer browsing along linear forest edges. *Northeastern Naturalist* 22: 262-272. doi:

Pinard, V., Dussault, C., Ouellet, J.P., Fortin, D., Courtois, R. (2012). Calving rate, calf survival rate, and habitat selection of forest‐dwelling caribou in a highly managed landscape. *The Journal of Wildlife Management* 76: 189-199. doi:

Pitman, J.W., Cain Iii, J.W., Liley, S.G., Gould, W.R., Quintana, N.T., Ballard, W.B. (2014). Post‐parturition habitat selection by elk calves and adult female elk in New Mexico. *The Journal of Wildlife Management* 78: 1216-1227. doi:

Proffitt, K.M., Grigg, J.L., Hamlin, K.L., Garrott, R.A. (2009). Contrasting effects of wolves and human hunters on elk behavioral responses to predation risk. *The Journal of Wildlife Management* 73: 345-356. doi:

Prokopenko, C.M., Boyce, M.S., Avgar, T. (2017). Extent-dependent habitat selection in a migratory large herbivore: road avoidance across scales. *Landscape Ecology* 32: 313-325. doi:

Pudyatmoko, S. (2018). Spatiotemporal inter-predator and predator–prey interactions of mammalian species in a tropical savanna and deciduous forest in Indonesia. *Mammal Research* 64: 1-12. doi:

Richard, J.H., Côté, S.D. (2016). Space use analyses suggest avoidance of a ski area by mountain goats. *The Journal of Wildlife Management* 80: 387-395. doi:

Riginos, C., Grace, J.B. (2008). Savanna tree density, herbivores, and the herbaceous community: bottom‐up vs. top‐down effects. *Ecology* 89: 2228-2238. doi:

Riginos, C. (2015). Climate and the landscape of fear in an African savanna. *Journal of Animal Ecology* 84: 124-133. doi:

Ripple, W.J., Beschta L.R. (2003). Wolf reintroduction, predation risk, and cottonwood recovery in Yellowstone National Park. *Forest Ecology and Management* 184: 299-313. doi:

Ripple, W.J., Beschta, R.L. (2004). Wolves, elk, willows, and trophic cascades in the upper Gallatin Range of Southwestern Montana, USA. *Forest Ecology and Management* 200: 161-181. doi:

Ripple, W.J., Beschta, R.L. (2006). Linking wolves to willows via risk-sensitive foraging by ungulates in the northern Yellowstone ecosystem. *Forest Ecology and Management* 230: 96-106. doi:

Ripple, W.J., Beschta, R.L. (2006). Linking a cougar decline, trophic cascade, and catastrophic regime shift in Zion National Park. *Biological Conservation* 133: 397-408. doi:

Ripple, W.J., Beschta RL. (2007). Restoring Yellowstone’s aspen with wolves. *Biological Conservation* 138: 514-519. doi:

Ripple, W.J., Beschta RL. (2008). Trophic cascades involving cougar, mule deer, and black oaks in Yosemite National Park. *Biological Conservation* 141: 1249-1256. doi:

Ripple, W.J., Beschta RL. (2012). Trophic cascades in Yellowstone: the first 15 years after wolf reintroduction. *Biological Conservation* 145: 205-213. doi:

Ripple, W.J., Beschta RL. Fortin, J.K., Robbins, C.T. (2014). Trophic cascades from wolves to grizzly bears in Yellowstone. *Journal of Animal Ecology* 83: 223-233. doi:

Ripple, W.J., Beschta, R.L., Painter, L.E. (2015). Trophic cascades from wolves to alders in Yellowstone. *Forest Ecology and Management* 354: 254-260. doi:

Robinson, B.G., Merrill, E.H. (2013). Foraging–vigilance trade-offs in a partially migratory population: comparing migrants and residents on a sympatric range. *Animal Behaviour* 85: 849-856. doi:

Rogala, J.K., Hebblewhite, M., Whittington, J., White, C.A., Coleshill, J., Musiani, M. (2011). Human activity differentially redistributes large mammals in the Canadian Rockies National Parks. *Ecology and Society* 16. doi:

Ross, J., Hearn, A.J., Johnson, P.J., Macdonald, D.W. (2013). Activity patterns and temporal avoidance by prey in response to Sunda clouded leopard predation risk. *Journal of Zoology* 290: 96-106. doi:

Sahlén, E., Noell, S., DePerno, C.S., Kindberg, J., Spong, G., Cromsigt, J.P. (2016). Phantoms of the forest: legacy risk effects of a regionally extinct large carnivore. *Ecology and Evolution* 6: 791-799. doi:

Saïd, S., Tolon, V., Brandt, S., Baubet, E. (2012). Sex effect on habitat selection in response to hunting disturbance: the study of wild boar. *European Journal of Wildlife Research* 58: 107-115. doi:

Samelius, G., Andrén, H., Kjellander, P., Liberg, O. (2013). Habitat selection and risk of predation: re-colonization by lynx had limited impact on habitat selection by roe deer. *PLoS One* 8: e75469. doi:

Sand, H., Wikenros, C., Wabakken, P., Liberg, O. (2006). Cross-continental differences in patterns of predation: will naive moose in Scandinavia ever learn?. *Proceedings of the Royal Society of London B: Biological Sciences* 273: 1421-1427. doi:

Sarmento, W.M., Berger, J. (2017). Human visitation limits the utility of protected areas as ecological baselines. *Biological Conservation* 212: 316-326. doi:

Sawyer, H., Nielson, R.M., Lindzey, F., McDonald, L.L. (2006). Winter habitat selection of mule deer before and during development of a natural gas field. *The Journal of Wildlife Management* 70: 396-403. doi:

Seamans, T.W., Blackwell, B.F., Linnell, K.E. (2016). Use of predator hair to enhance perceived risk to white-tailed deer in a foraging context. *Human–Wildlife Interactions* 10: 300–311. doi:

Schuttler, S.G., Parsons, A.W., Forrester, T.D., Baker, M.C., McShea, W.J., Costello, R., Kays, R. (2017). Deer on the lookout: how hunting, hiking and coyotes affect white‐tailed deer vigilance. *Journal of Zoology* 301: 320-327. doi:

Semeniuk, C.A., Musiani, M., Hebblewhite, M., Grindal, S., Marceau, D.J. (2012). Evaluating risk effects of industrial features on woodland caribou habitat selection in west central Alberta using agent-based modelling. *Procedia Environmental Sciences* 13: 698. doi:

Semeniuk, C.A., Musiani, M., Birkigt, D.A., Hebblewhite, M., Grindal, S., Marceau, D.J. (2014). Identifying non-independent anthropogenic risks using a behavioral individual-based model. *Ecological Complexity* 17: 67-78. doi:

Shamoon, H., Dayan, T., Saltz, D. (2017). Cattle grazing effects on mountain gazelles in Mediterranean natural landscapes. *The Journal of Wildlife Management* 81: 1351-1362. doi:

Shamoon, H., Maor, R., Saltz, D., Dayan, T. (2018). Increased mammal nocturnality in agricultural landscapes results in fragmentation due to cascading effects. *Biological Conservation* 226: 32-41. doi:

Shannon, G., Cordes, L.S., Hardy, A.R., Angeloni, L.M., Crooks, K.R. (2014). Behavioral responses associated with a human-mediated predator shelter. *PLoS One* 9: e94630. doi:

Shelton, A.L., Henning, J.A., Schultz, P., Clay, K. (2014). Effects of abundant white-tailed deer on vegetation, animals, mycorrhizal fungi, and soils. *Forest Ecology and Management* 320: 39-49. doi:

Silva-Rodríguez, E.A., Sieving K.E. (2012). Domestic dogs shape the landscape-scale distribution of a threatened forest ungulate. *Biological Conservation* 150: 103-110. doi:

Sivertsen, T.R., Åhman, B., Steyaert, S.M., Rönnegård, L., Frank, J., Segerström, P., Støen, O., Skarin, A. (2016). Reindeer habitat selection under the risk of brown bear predation during calving season. *Ecosphere* 7: e01583. doi:

Sönnichsen, L., Bokje, M., Marchal, J., Hofer, H., Jędrzejewska, B., Kramer‐Schadt, S., Ortmann, S. (2013). Behavioural Responses of European Roe Deer to Temporal Variation in Predation Risk. *Ethology* 119: 233-243. doi:

Stears, K., Shrader, A.M. (2015). Increases in food availability can tempt oribi antelope into taking greater risks at both large and small spatial scales. *Animal Behaviour* 108: 155-164. doi:

Stillfried, M., Gras, P., Börner, K., Göritz, F., Painer, J., Röllig, K., Wenzler, M., Hofer, H., Ortmann, S., Kramer-Schadt, S. (2017). Secrets of success in a landscape of fear: Urban wild boar adjust risk perception and tolerate disturbance. *Frontiers in Ecology and Evolution* 5: 157. doi:

Støen, O.G., Neumann, W., Ericsson, G., Swenson, J.E., Dettki, H., Kindberg, J., Nellemann, C. (2010). Behavioural response of moose *Alces alces* and brown bears *Ursus arctos* to direct helicopter approach by researchers. *Wildlife Biology* 16: 292-300. doi:

Sullivan, J.D., Ditchkoff, S.S., Collier, B.A., Ruth, C.R., Raglin, J.B. (2018). Recognizing the danger zone: response of female white-tailed to discrete hunting events. *Wildlife Biology* wlb-00455. doi: 10.2981/wlb.00455

Sundararaj, V., McLaren, B.E., Morris, D.W., Goyal, S.P. (2012). Can rare positive interactions become common when large carnivores consume livestock?. *Ecology* 93: 272-280. doi:

Sunde, P., Olesen, C.R., Madsen, T.L., Haugaard, L. (2009). Behavioural responses of GPS-collared female red deer *Cervus elaphus* to driven hunts. *Wildlife Biology* 15: 454-460. doi:

Tadesse, S.A., Kotler, B.P. (2011). Seasonal habitat use by Nubian ibex (*Capra nubiana*) evaluated with behavioral indicators. *Israel Journal of Ecology & Evolution* 57: 223-246. doi:

Tadesse, S.A., Kotler, B.P. (2012). Impact of tourism on Nubian Ibex (*Capra nubiana*) revealed through assessment of behavioral indicators. *Behavioral Ecology* 23: 1257-1262. doi:

Tambling, C.J., Druce, D.J., Hayward, M.W., Castley, J.G., Adendorff, J., Kerley, G.I. (2012). Spatial and temporal changes in group dynamics and range use enable anti‐predator responses in African buffalo. *Ecology* 93: 1297-1304. doi:

Tambling, C.J., Minnie, L., Meyer, J., Freeman, E.W., Santymire, R.M., Adendorff, J., Kerley, G.I. (2015). Temporal shifts in activity of prey following large predator reintroductions. *Behavioral Ecology and Sociobiology* 69: 1153-1161. doi:

Teichman, K.J., Nielsen, S.E., Roland, J. (2013). Trophic cascades: linking ungulates to shrub‐dependent birds and butterflies. *Journal of Animal Ecology* 82: 1288-1299. doi:

Thaker, M., Vanak, A.T., Owen, C.R., Ogden, M.B., Niemann, S.M., Slotow, R. (2011). Minimizing predation risk in a landscape of multiple predators: effects on the spatial distribution of African ungulates. *Ecology* 92: 398-407. doi:

Theuerkauf, J., Rouys S. (2008). Habitat selection by ungulates in relation to predation risk by wolves and humans in the Białowieża Forest, Poland. *Forest Ecology and Management* 256: 1325-1332. doi:

Thurfjell, H., Spong, G., Ericsson, G. (2013). Effects of hunting on wild boar *Sus scrofa* behaviour. *Wildlife Biology* 19: 87-93. doi:

Thurfjell, H., Ciuti, S., Boyce, M.S. (2017). Learning from the mistakes of others: How female elk (*Cervus elaphus*) adjust behaviour with age to avoid hunters. *PloS one* 12: e0178082. doi:

Tolon, V., Dray, S., Loison, A., Zeileis, A., Fischer, C., Baubet, E. (2009). Responding to spatial and temporal variations in predation risk: space use of a game species in a changing landscape of fear. *Canadian Journal of Zoology* 87: 1129-1137. doi:

Torres, R.T., Santos, J., Linnell, J.D., Virgós, E., Fonseca, C. (2011). Factors affecting roe deer occurrence in a Mediterranean landscape, Northeastern Portugal. *Mammalian Biology-Zeitschrift für Säugetierkunde* 76: 491-497. doi:

Valeix, M., Loveridge, A.J., Chamaillé-Jammes, S., Davidson, Z., Murindagomo, F., Fritz, H., Macdonald, D.W. (2009). Behavioral adjustments of African herbivores to predation risk by lions: spatiotemporal variations influence habitat use. *Ecology* 90: 23-30. doi:

van Beeck Calkoen, S.T., Kuijper, D.P., Sand, H., Singh, N.J., van Wieren, S.E., Cromsigt, J.P. (2018). Does wolf presence reduce moose browsing intensity in young forest plantations?. *Ecography* 41: 1776-1787. doi:

Van der Wal, R., Irvine, J., Stien, A., Shepherd, N., Albon, S.D. (2000). Faecal avoidance and the risk of infection by nematodes in a natural population of reindeer. *Oecologia* 124: 19-25. doi:

van Ginkel, H.A.L., Kuijper, D.P.J., Schotanus, J., Smit, C. (2018). Wolves and Tree Logs: Landscape-Scale and Fine-Scale Risk Factors Interactively Influence Tree Regeneration. *Ecosystems* 1-11. doi:

Vijayan, S., Morris, D.W., McLaren B.E. (2012). Prey habitat selection under shared predation: tradeoffs between risk and competition?. *Oikos* 121: 783-789. doi:

Villepique, J.T., Pierce, B.M., Bleich, V.C., Andic, A., Bowyer, R.T. (2015). Resource Selection by an endangered ungulate: a test of predator-induced range abandonment. *Advances in Ecology* 2015: 357080. doi:

Visscher, D.R., Macleod, I., Vujnovic, K., Vujnovic, D., Dewitt, P.D. (2017). Human risk induced behavioral shifts in refuge use by elk in an agricultural matrix. *Wildlife Society Bulletin* 41: 162-169. doi:

Wakefield, S., Attum, O. (2006). The effects of human visits on the use of a waterhole by endangered ungulates. *Journal of Arid Environments* 65: 668-672. doi:

Wallach, A.D., Johnson, C.N., Ritchie, E.G., O’Neill, A.J. (2010). Predator control promotes invasive dominated ecological states. *Ecology Letters* 13: 1008-1018. doi:

Waser, N.M., Price, M.V., Blumstein, D.T., Arózqueta, S.R., Escobar, B.D.C., Pickens, R., Pistoia, A. (2014). Coyotes, deer, and wildflowers: diverse evidence points to a trophic cascade. *Naturwissenschaften* 101: 427-436. doi:

Weir, J.N., Mahoney, S.P., McLaren, B., Ferguson, S.H. (2007). Effects of mine development on woodland caribou *Rangifer tarandus* distribution. *Wildlife Biology* 13: 66-74. doi:

White, C.A., Feller, M.C., Bayley, S. (2003). Predation risk and the functional response of elk–aspen herbivory. *Forest Ecology and Management* 181: 77-97. doi:

White, P.J., Garrott, R.A., Hamlin, K.L., Cook, R.C., Cook, J.G., Cunningham, J.A. (2011). Body condition and pregnancy in northern Yellowstone elk: Evidence for predation risk effects?. *Ecological Applications* 21: 3-8. doi:

White, K.S., Gregovich, D.P. (2017). Mountain goat resource selection in relation to mining-related disturbance. *Wildlife Biology* wlb-00277. doi:

Wikenros, C., Kuijper, D.P., Behnke, R., Schmidt, K. (2015). Behavioural responses of ungulates to indirect cues of an ambush predator. *Behaviour* 152: 1019-1040. doi:

Wikenros, C., Balogh, G., Sand, H., Nicholson, K.L., Månsson, J. (2016). Mobility of moose—comparing the effects of wolf predation risk, reproductive status, and seasonality. *Ecology and Evolution* 6: 8870-8880. doi:

Witter, L.A., Johnson, C.J., Croft, B., Gunn, A., Gillingham, M.P. (2012). Behavioural trade‐offs in response to external stimuli: time allocation of an Arctic ungulate during varying intensities of harassment by parasitic flies. *Journal of Animal Ecology* 81: 284-295. doi:

Wolff, J.O., Horn, T.V. (2003). Vigilance and foraging patterns of American elk during the rut in habitats with and without predators. *Canadian Journal of Zoology* 81: 266-271. doi:

Yan, W.B., Zeng, Z.G., Gong, H.S., He, X.B., Liu, X.Y., Si, K.C., Song, Y.L. (2017). Habitat use and selection by takin in the Qinling Mountains, China. *Wildlife Research* 43: 671-680. doi:

Zbyryt, A., Bubnicki, J.W., Kuijper, D.P., Dehnhard, M., Churski, M., Schmidt, K. (2017). Do wild ungulates experience higher stress with humans than with large carnivores?. *Behavioral Ecology* 29: 19-30. doi:

Zwijacz-Kozica, T., Selva, N., Barja, I., Silván, G., Martínez-Fernández, L., Illera, J.C., Jodłowski, M. (2013). Concentration of fecal cortisol metabolites in chamois in relation to tourist pressure in Tatra National Park (South Poland). *Acta Theriologica* 58: 215-222. doi:
